# Human Neutrophils Produce De Novo Thyroid Hormones Mediated by the Induction of the Oxidative Burst

**DOI:** 10.3390/biom16081081

**Published:** 2026-07-23

**Authors:** Mahmood Y. Bilal, Umida Ganieva, Thanh Luu, Joanne Kwak-Kim, Svetlana Dambaeva

**Affiliations:** 1 Clinical Immunology Laboratory, Rosalind Franklin University of Medicine and Science, North Chicago, IL 60064, USA; 2 Center for Cancer Cell Biology, Immunology, and Infection, Rosalind Franklin University of Medicine and Science, North Chicago, IL 60064, USA; 3 Reproductive Medicine and Immunology, Obstetrics and Gynecology, Clinical Sciences Department, Chicago Medical School, Rosalind Franklin University of Medicine and Science, North Chicago, IL 60064, USA

**Keywords:** extrathyroidal thyroxine, MPO, neutrophil iodination, oxidative burst, iodine

## Abstract

Thyroid hormone synthesis involves several key steps culminating in the organification of iodide into iodothyronines, thyroxine, and triiodothyronine. To date, there is no clear mechanistic evidence of extrathyroidal iodothyronine synthesis. Upon iodine (KI/I_2_) loading, human neutrophils produced significant amounts of de novo iodothyronines as determined by diagnostic immunoassay and LCMS/MS. Treatment of neutrophils with sodium iodide resulted in a lower hormone yield, which was enhanced following the addition of copper chloride to culture media. The appearance of thyroid hormones was observed only after induction of the oxidative burst with PMA. Synthesis of hormones was abolished by the nicotinamide adenine dinucleotide phosphate (NADPH) oxidase inhibitor, DPI. Essential components for iodothyronine synthesis included hydrogen peroxide, myeloperoxidase, and either serum or thyroglobulin serving as an iodination substrate. In a series of novel experiments, we show a serine protease requirement for liberation of thyroid hormones from reservoirs of iodinated proteins. Serine proteases, proteinase K, and neutrophil-derived elastase, but not cysteine protease cathepsin B, released thyroxine from in vitro iodinated or native thyroid-derived thyroglobulin, respectively. Our studies show that neutrophil-mediated iodothyronine synthesis is an extracellular, multi-step, enzymatic process initiated upon cellular activation, but is independent of neutrophil viability window. Overall, this report presents a series of cellular and enzymatic experiments demonstrating the induction of thyroid hormones by non-classical mechanisms that can potentially regulate localized levels of iodothyronines.

## 1. Introduction

Thyroid-derived iodothyronines, thyroxine (T_4_) and triiodothyronine (T_3_), play essential roles in tissue metabolism and are crucial for the proper function of immune cells [1,2,3,4]. These molecules are required for optimal pregnancy and are indispensable for normal brain and child development [4,5,6,7]. Assembly of thyroid hormones occurs primarily within the thyroid gland, beginning with the transport of iodide into thyroid follicular cells and the colloid-utilizing sodium iodide symporter (NIS) and PENDRIN, respectively [1,5]. Conjugation of iodide within thyroglobulin (TG) in the thyroid colloid requires the presence of hydrogen peroxide (H_2_O_2_) generated by NADPH oxidase dual oxidase (DUOX). Whilst superoxide is generated from NADPH oxidase activation, it acts as a precursor to H_2_O_2_, which is produced through a dismutase reaction of superoxide [8,9]. These oxidants are thence utilized by thyroid peroxidase (TPO), whereby iodide is oxidized into iodine, followed by conjugation onto tyrosyl residues present on TG to form mono- or di-iodotyrosine (MIT/DIT) [1,5]. The latter is then formed into T_4_ or T_3_ via a coupling reaction that involves peroxidase activity [10,11]. Finally, proteolytic degradation of TG in the thyroid results in the release of mostly pro-hormone T_4_ (approximately 80–90%) and its more active form, T_3_, into circulation [5]. Degradation of TG has been shown to occur in part through the action of cathepsin’s protease activity [12]. To this end, our previous work demonstrated that human peripheral blood leukocytes can proteolytically degrade native thyroid-derived human TG, resulting in release of T_4_/T_3_ into the supernatant [5].

Evidence for an unclear extrathyroidal source of thyroid hormones has been suggested as early as 1967, based on detectable T_4_ levels in thyroidectomized rats administered a 5 mg daily dose of iodide [13]. In 2011, Nagao et al. confirmed these observations using LCMS. The author suggested the presence of an unknown extrathyroidal source of synthesis [14,15]. Nevertheless, the potential and role of a secondary “self-sufficient” extrathyroidal thyroid-hormone forming machinery is not well understood. Indeed, there needs to be a requirement for a TPO-like enzyme, in addition to extrathyroidal iodination sites. Extrathyroidal hormone machinery will likely also require iodide-concentrating receptors, complemented by the deiodinase (DIO) family of enzymes for increasing the concentration of localized active hormone, T_3_. With regard to iodide transporters, they are widely expressed and can be utilized by different cell types to regulate the transcriptome [5,16,17]. We found that immune cells upregulated iodide receptors upon activation and displayed marked production of cytokines upon treatment with NaI or Lugol’s iodine [5]. These mechanisms were shown to be independent of de novo thyroid hormone synthesis in peripheral blood mononuclear cells (PBMC).

Next, studies in the era of the 1930s and 1940s, including the work of Ludwig and von Mutzenbecker in 1939, showed the iodination potential of several proteins, such as casein, serum albumin, and serum globulins [18,19]. Preparations of these proteins with iodine under careful experimental conditions yielded substances that exhibited thyroid hormone activity. Indeed, Harington and Rivers et al. obtained a crystalline thyroxine from iodinated casein protein [20]. Others showed that iodination of serum proteins produced a substance that gave successful responses against myxedema and cretinism in thyroidectomized humans and rabbits [21]. Similar observations were found when thyroidectomized goats were given iodinated casein protein [22]. Nevertheless, to our knowledge, there is still no clear demonstration of complete sequential synthesis of de novo bioavailable thyroxine in humans by extrathyroidal machinery. If this indeed occurs, then one could postulate that iodo-nucleation sites could include TG, albumin, or globulins due to presence in non-thyroidal tissues [5,18,23,24]. In the case of TG, it can be found in the plasma of healthy individuals, with higher levels observed in those with hypothyroidism and/or iodine deficiency [25,26,27]. Albumin is less understood in the context of thyroid hormone synthesis. As early as 1911, there was evidence of the iodination potential of albumin, which resulted in the formation of DIT [19]. Experiments in 1976 detected T_4_ and T_3_ in thyroid-derived albumin, demonstrating that TG may not be the only iodination site for hormone synthesis within the thyroid gland [23].

TPO is considered to be the primary thyroidal peroxidase responsible for the iodo-conjugation of TG [11,28]. TPO is primarily limited in expression to the thyroid glands, indicating that any potential for extrathyroidal hormone formation is likely mediated by other peroxidases, such as myeloperoxidase (MPO). MPO is the most abundant peroxidase in humans and has similar enzymatic function to TPO, eosinophil-peroxidase (EPO), and lactoperoxidase (LPO) [8,29]. MPO can be released extracellularly, and neutrophils are its major reservoir, whereby MPO constitutes 2–5% of the neutrophil’s mass [30,31]. MPO and TPO have stark similarities in terms of nucleotide homology and resultant enzymatic reactions [28,29]. The functional protein site between MPO and TPO has 58% sequence homology, with the heme active site showing 74% sequence similarity. In the thyroid, TPO’s oxidation of iodide results in thyroid hormone production. In neutrophils, MPO functions as a microbicide because of the resultant products of its halogenation cycle. Specifically, upon neutrophil activation, oxidation of halogens yields antimicrobial hypohalous acids, primarily hypochlorous acid, that play an important role in clearing bacterial infections [8]. Although chloride is the primary halogen for MPO due to its abundance in plasma, oxidation of iodide also produces a strong antimicrobial agent, hypoiodite (HOI). Iodide is more easily oxidized, wherein MPO does not require millimolar range concentrations, as in the case of chloride [32]. Logically, given the neutrophil’s capacity to produce H_2_O_2_ via NADPH oxidase, the neutrophil becomes a prime candidate for potential thyroid hormone synthesis, particularly because of its high MPO content. In addition, our work and others show that neutrophils are able to receive iodide into the cellular compartment [5,33]. This includes recent work by Foti et al., who demonstrated the presence of iodo-proteins within activated human neutrophils [34].

Experimental evidence pointing toward the potential role of MPO in thyroid hormone synthesis was demonstrated via in vitro enzymatic studies by Yip et al. in 1963, wherein MPO, derived from dog pus, catalyzed the coupling of DIT to produce compounds indistinguishable from thyroxine [35]. Others have shown that both LPO and horseradish peroxidase perform similar functions in terms of thyroxine formation from DIT [28,35]. In 1992, Taurog et al. showed that human MPO can iodinate TG derived from a large goitrous human thyroid in the presence of H_2_O_2_ [28]. Taurog et al. further demonstrated that TG pre-labeled with radioactive DIT underwent coupling after incubation with MPO; however, the increase in T_4_ was minimal (from basal 1.5% to 11%). The iodination of TG by MPO in vitro indicates a potential for involvement of in vivo extrathyroidal thyroid hormone synthesis by its main host, the neutrophil [28].

In terms of evidence for extrathyroidal T_4_ synthesis in human cellular systems, Victor Stolc’s interesting work in 1971 provides, thus far, one of the most compelling experiments [36]. Utilizing radioiodide in stimulated leukocytes resulted in the appearance of MIT, DIT, and a “thyroxine-like” compound. However, Stolc raises the possibility that this observed T_4_ may not be a result of de novo leukocyte synthesis. Therefore, although in vitro and ex vivo studies show the potential involvement of leukocytes, MPO, TG, and albumin in extrathyroidal thyroid hormone production, the observations are fragmented. Specifically, there is no clear demonstrated mechanism of de novo release of thyroxine from non-iodinated proteins ex vivo. One exception is the work by Banerjee et al., who demonstrated that mouse stomach and submaxillary glands could concentrate iodide and produce MIT/DIT, but there was no detectable thyroid hormone. They found, however, that thyroxine only formed when TG was utilized in an in vitro assay utilizing soluble stomach protein preparation (i.e., non-cellular assay) [37]. Overall, outside of these in vitro studies, demonstration of de novo extrathyroidal thyroid hormone synthesis by human cells, followed by release of a biologically relevant quantity of T_4_ and T_3_, has not been clearly shown.

In this study, which we present here as a proof of concept, we attempted to elucidate whether human cellular systems, outside of the thyroid, could produce de novo iodothyronines. We show that human neutrophils possess the capacity to produce both T_4_ and T_3_, as evaluated by our controlled ex vivo conditions. The presence of iodothyronines was confirmed by four commonly utilized diagnostic immunoassays and further verified by LCMS/MS. Our observations suggest important mechanisms by which extrathyroidal hormones are produced, namely the requirement for MPO, H_2_O_2_-producing systems, and proteolytic enzymes.

## 2. Materials and Methods

### 2.1. Isolation of Human Neutrophils and PBMC

Whole blood was drawn in sodium heparin. The consent and documentation process associated with these donors was approved by the IRB of Rosalind Franklin University of Medicine and Science. Neutrophils were isolated per protocol of Nauseef et al., utilizing 3% dextran (Sigma-Aldrich, 31392, St. Louis, MO, USA) enrichment followed by Ficoll-Paque [38]. For PBMC extraction, the Ficoll-Paque resultant middle interface (lighter band) was carefully collected, followed by PBS wash. Red blood cells were removed from the neutrophil pellet by gentle water lysis (20 s), followed by tonicity adjustment with physiological saline. Isolated neutrophils were washed in Hank’s Balanced Salt Solution (HBSS), without calcium or magnesium, prior to incubation in culture media. Purity of neutrophils was determined using flow cytometry analysis (BD Lyric, Franklin Lakes, NJ, USA). Neutrophils were stained with CD16 (BD, 338433) and CD66b (BD, 561927). Double-positive cells were calculated across five donors, with a mean purity of 92.5% (standard deviation, 2.2%).

### 2.2. Induction of Thyroid Hormone Synthesis

Neutrophils were cultured at 37 °C and 5% CO_2_ in complete DMEM/F12 medium (ThermoFisher, 11320033, Waltham, MA, USA) supplemented with varying concentrations of non-heat-inactivated, charcoal-stripped fetal bovine serum (FBS) (ThermoFisher, A3382101). Treatment of neutrophils included the following reagents: Lugol’s iodine solution (Sigma-Aldrich, 32922), sodium iodide (NaI) (Sigma-Aldrich, 383112), human albumin (Sigma-Aldrich, 126654), recombinant human albumin (Albumin Bioscience, 1001, Huntsville, AL, USA), diphenyleneiodonium (DPI, MCE, HY-100965, Monmouth Junction, NJ, USA), phorbol 12-myristate 13-acetate (PMA, MCE, HY-18739), lipopolysaccharides (LPS, MCE, HY-D1056), and tumor necrosis factor-alpha (TNFα, MCE, HY-P7058). Concentrations utilized throughout the study are as follows: PMA (2.5 µg/mL), LPS (10 ng/mL), TNFα (20 ng/mL), Lugol’s iodine (500–700 µM), and NaI (500–1000 µM).

For pan T cell activation, PBMCs were loaded on 24-well plates coated with anti-CD3, as previously described [39]. Briefly, cells were suspended in above media at a concentration of 1 × 10^6^/mL, with or without Lugol’s iodine solution. The cells were then plated on wells coated with 1 µg/mL anti-CD3 (Biolegend clone OKT3, 317302, San Diego, CA, USA) and 0.5 µg/mL soluble anti-CD28 (Biolegend clone CD28.2, 302902). K562 cell line (ATCC, CCL-243, Manassas, VA, USA) was propagated in similar media.

Human recombinant TG (Hytest, 8RTG4, Turku, Finland) was derived from a mammalian cell line and yields a protein of 304.640 kDa. Lyophilized TG was resuspended at 1 mg/mL with deionized water. In contrast to thyroid gland-derived TG, recombinant TG is expected to be non-iodinated. The manufacturer confirmed that expression of recombinant TG was in a medium without serum and with only trace amounts of iodide. Our immunoassay analysis showed undetectable thyroid hormone signal within Hytest recombinant TG preparations.

### 2.3. Serum Thyroid Hormone Analysis—Chemiluminescence Immunoassay

Thyroid hormones were evaluated using an in vitro diagnostics (IVD)-based chemiluminescent immunoassay analyzer, access-2 (Beckman Coulter, Brea, CA, USA), per manufacturer’s recommendations. The assays include total T_4_ (TT_4_, Beckman Coulter, 33800), total T_3_ (TT_3_, Beckman Coulter, 33830), free T_4_ (fT4, Beckman Coulter, 33880), and free T_3_ (fT3, Beckman Coulter, A13422). Data represented are values after subtraction of background signals for each cellular treatment. Background signal for DMEM/F12 media was either undetectable or below/near the limits of quantification (LOQ). Data points with negative values after background adjustment were converted to “0.”

Analysis of non-diluted charcoal-stripped FBS from different lots showed undetectable levels, or levels below the assay’s LOQ for all analytes. Of note, certain levels of biotin in culture media, such as RPMI, can cause interference with the access-2 immunoassay system. Evaluation of RPMI media showed high interference for fT_3_. In contrast, DMEM/F12 media used in this study showed no detectable signals or signals lower than the LOQ.

Per manufacturer’s (Beckman Coulter) interference and analytical sensitivity studies, the LOQ for TT_4_, TT_3_, fT_4_, and fT_3_ is 0.50 µg/dL, 0.1 ng/mL, 0.25 ng/dL, and 0.88 pg/mL, respectively. The fT_3_ assay has <0.5% cross-reactivity with analytes: reverse T_3_ (rT_3_) tetraiodothyroacetic acid, D-thyroxine, L-thyroxine, 3,5 diiodothyronine, diiodo-L-tyrosine, and monoiodotyrosine. The fT_4_, TT_3_, and TT_4_ assays have similar cross-reactivity properties, including for MIT/DIT. Cross-reactivity reports for each assay are available upon request.

### 2.4. Liquid Chromatography (LC) Tandem Mass Spectrometry (MS)

LCMS/MS for T_4_/T_3_ was performed at the High-Resolution Mass Spectrometry Facility (HRMSF, University of Iowa). Quantitative detection of thyroid hormones used external and internal (carbon-13) standards for T_4_ and T_3_. The standards were obtained from Sigma-Aldrich as follows: L-Thyroxine (T_4_) solution/T073, [3,3′,5-Triiodo-L-thyronine] (T_3_) solution/T074, L-Thyroxine-^13^C_6_ solution/T076, and [3,3′,5-Triiodo-L-thyronine-^13^C_6_] solution/T077. Hormone extraction from cell culture was performed using 200 µL of the sample with an internal carbon-13 reference standard. Then, 400 µL of acetonitrile (Fisher LC/MS optima, A955) was added to the sample/standard mixture, followed by centrifugation (13,000 rpm, 10 min, 4 °C). The supernatant was transferred to a fresh tube, and the solvent was evaporated under nitrogen. Samples were then reconstituted with 200 µL 3:1 water/methanol. LCMS/MS data were acquired using a Waters Xevo TQ-S cronos triple quadrupole mass spectrometer (Waters Corporation, Milford, MA, USA) coupled to a Waters Acquity H-Class UPLC system. Waters MassLynx 4.2 software was used for data acquisition and initial data quality control. Further data processing was performed with Waters TargetLynx for peak detection and quantification.

Detailed information on standard calibrations/reconstitutions, mobile phase, and other LCMS/MS parameters can be found under File S1.

### 2.5. Thyroid Hormone Liberation from In Vitro MPO-Mediated Iodination Assay

MPO derived from human neutrophils was obtained from (Athens Bioscience, 16-14-130,000, Athens, GA, USA)). Different conditions were evaluated as follows: MPO was resuspended at 45 or 50 µg/mL in DMEM/F12 media containing 500 or 700 µM of Lugol’s iodine. A mixture of FBS (5 or 7%) and/or TG (15 or 50 µg/mL) was added as an iodination substrate, followed by H_2_O_2_ (135 or 200 µM). A second tube utilized water instead of H_2_O_2_ as a control. A third enzymatic reaction containing only Lugol’s iodine and H_2_O_2_ was utilized as an MPO control. H_2_O_2_ or water was added last to the reaction tubes. The three reactions were then incubated at 37 °C for approximately 6 h.

For resting (un-activated) neutrophil co-incubation, 550 µL of each of the completed MPO enzymatic mixtures was allowed to resuspend cells for 24 h, after which the supernatants from non-cellular (original reaction media) and cleared-cellular (i.e., treated with resting neutrophil) were assayed for thyroid hormone liberation.

For protease (proteinase K)-mediated liberation of hormones, one condition was utilized as follows: MPO (50 µg/mL), Lugol’s iodine (700 µM), FBS (5%), TG (20 µg/mL), and H_2_O_2_ (200 µM). The in vitro MPO reaction was initiated as mentioned above and then stored at −80 °C. For proteolytic degradation, each reaction (550 µL) was split into a group that received 1.2 units of thermolabile proteinase K (NEB, P8111S) or HBSS as a control. The reaction was incubated at 30 °C for 3 h, followed by heat inactivation of the thermolabile protease at 56 °C for 25 min. Thyroid hormones were immediately evaluated via immunoassay, as described above.

### 2.6. Thyroid Hormone Liberation from Native Human Thyroglobulin

Human native TG (Bio-Rad, PHP236, Hercules, CA, USA) was diluted in 500 µL DMEM/F12 medium at 10 µg/mL, followed by addition of 1 unit of neutrophil-derived human elastase (Athens Bioscience, 16-14-051200) or 0.4 unit of cathepsin B (Athens Bioscience, 16-12-030102) derived from human liver. The reaction was incubated for 24 h. For cellular experiments, human neutrophils suspended at 9 × 10^6^/mL in DMEM/F12 media were incubated with human native TG at 10 µg/mL for 24 h. Thyroid hormones were immediately evaluated via immunoassay, as described above.

### 2.7. Statistical Analysis

Analysis of all data was performed using GraphPad Prism (version 11.0.0) and Microsoft Excel with a two-tailed *t*-test assuming normal distribution. Level of significance, *p* < 0.05 and *p* < 0.005, is presented in the appropriate figures as “*” and “**”, respectively. Unless otherwise stated, all graphs were created utilizing GraphPad Prism software. Model/schematic figures were created as follows:

- Figure 5: Created in BioRender. Bilal, M. (2026) https://BioRender.com/xne4fkf, accessed on 16 June 2026.
- Figure 9A: Created in BioRender. Bilal, M. (2026) https://BioRender.com/v8g6psh, accessed on 16 June 2026.
- Figure 9B: Created in BioRender. Bilal, M. (2026) https://BioRender.com/3c52z4p, accessed on 16 June 2026.

## 3. Results

### 3.1. Preliminary Experiments and Design of Optimal Extracellular Milieu for Thyroid Hormone Synthesis

It is important to first describe the initial exploratory experiments leading to our choice of the human neutrophil as a candidate for extrathyroidal thyroid hormone synthesis. Our previous work demonstrated that human leukocytes harbor iodide receptors, along with the capacity to digest naturally iodinated TG from human thyroid gland to produce T_4_ and T_3_ [5]. The latter is the final step within the thyroid glands required for T_4_ and T_3_ production. This was cell-dependent, as Jurkat T cells could not produce this effect [5].

**Figure 1:**
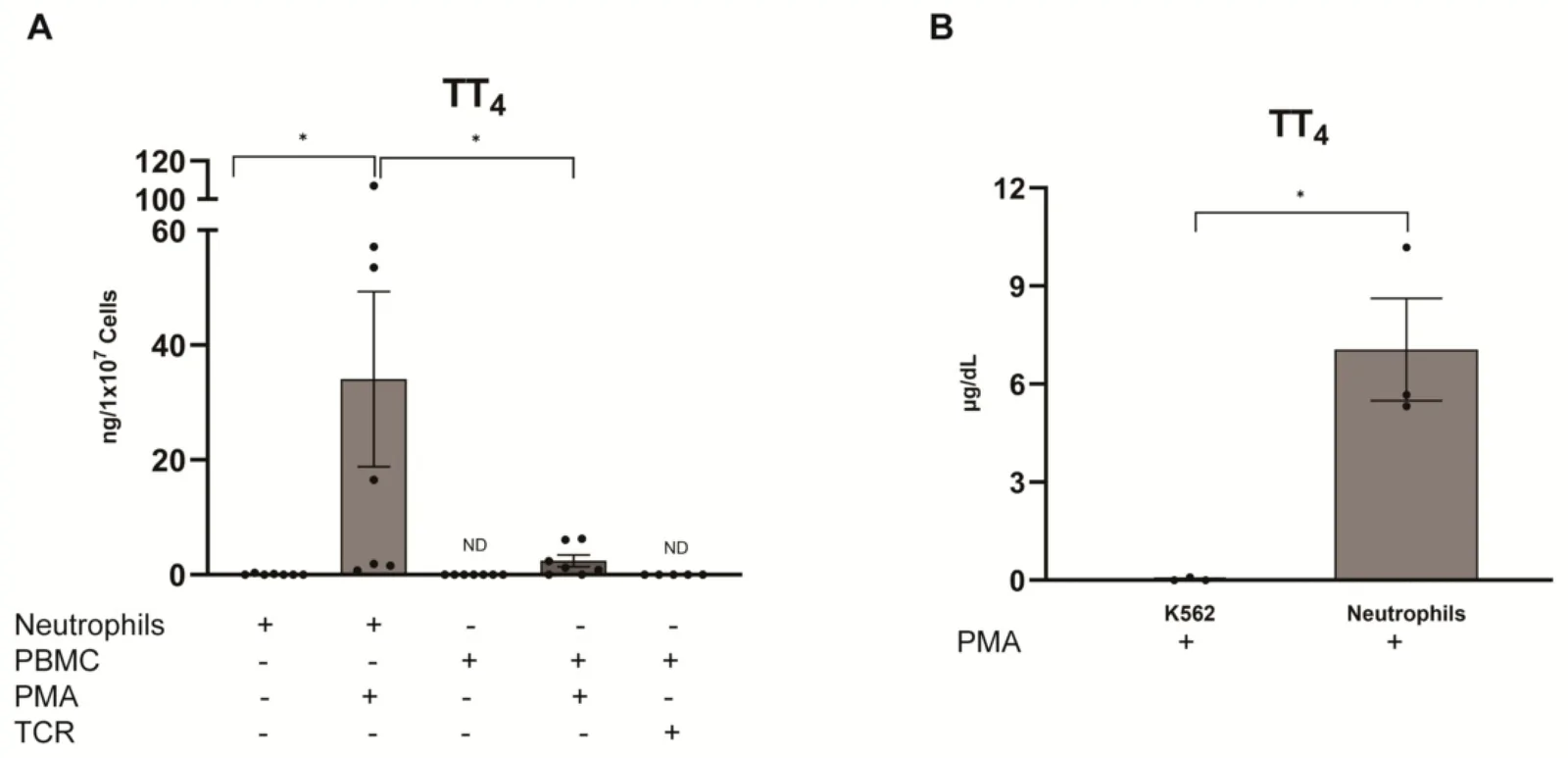
Representative preliminary experiments leading to choice of neutrophils as a cellular model for thyroid hormone synthesis. (**A**) Neutrophils or PBMC from the same seven donors were left unstimulated or activated (PMA) in 7% FBS with 700 µM of iodine (KI/I_2_) for 48 h. Alternatively, another set of five PBMC donors was activated for 3 days with plate-bound anti-CD3/anti-CD28 (TCR), as described in Methods section. (**B**) K562 cell line was compared to three neutrophil donors. Supernatant was evaluated for total T_4_ utilizing chemiluminescence immunoassay. Results are presented as mean ± SEM.

To understand whether or not immune cells could produce de novo thyroid hormone, we commenced a series of exploratory experiments. These experiments compared human-activated and non-activated PBMC, activated (pan) T cells, activated CD4+ T cells, and neutrophils. The experiments were performed in the presence of multiple variables, including nutrients and specific (or general) stimulants. Nutrients tested included iodine, iodide, zinc, copper, l-tyrosine, and increased concentration of glucose, with or without stimulatory signals either as PMA or anti-CD3/anti-CD28. Most of the experimental variables did not produce detectable T_4_ or T_3_ signals, except for small reproducible signals from serum-free media containing activated neutrophils, after addition of KI/I_2_. PBMCs exhibited variable but mostly inconsistent low-level, or undetectable, hormone signal. Results were confounded due to donor-to-donor variability in hormone signals compounded by the unknown optimal variables for hormone production. Results of some of these experimentations are shown, whereby neutrophils and PBMCs, from the same donors, were compared in terms of PMA activation (Figure 1A). In presence of KI/I_2_, activated neutrophils had a much higher T_4_ signal compared to PBMC. Another group of PBMC was activated through the T-cell receptor (TCR) pathway for 3 days, which resulted in no detectable levels of thyroid hormones (Figure 1A). T-cell receptor activation was evaluated by the substantial increase of interleukin-6 in culture supernatant (mean = 22.4 ng/mL). Similarly, evaluation of K562 cell lines stimulated with PMA had essentially undetectable T_4_ signal compared to human neutrophils (Figure 1B).

Our explorations concluded that neutrophils should be chosen as the immune cell candidate for producing iodothyronines at biologically significant levels. Indeed, a comparison of neutrophils’ enzymatic machinery and biochemical output with the thyroid gland further suggested the potential for neutrophil-mediated thyroid hormone synthesis (Table 1). Final results from our preliminary experiments showed that the optimal signal was in the presence of iodine (KI/I_2_) and DMEM/F12 media supplemented with 3–10% charcoal-stripped FBS. The presence of FBS proved to be an important component, as we shall detail in this report.

### 3.2. Activated Neutrophils Pulsed with Iodine Produce De Novo T_4_ and T_3_

Isolated human neutrophils were pulsed with KI/I_2_, with or without an activation signal (PMA), for indicated time points, and then thyroid hormones were probed via immunoassay. Both free and total fractions of thyroid hormones were evaluated in order to avoid “masking” of hormone release, as almost all of T_4_ and T_3_ is bound to serum proteins in normal human plasma (free fractions of 0.01–0.02% and 0.5%, respectively) [1]. Results were then normalized to neutrophil counts, given donor-to-donor variability in neutrophil yield. Analysis of data with absolute hormone concentration showed similar results.

**Figure 2:**
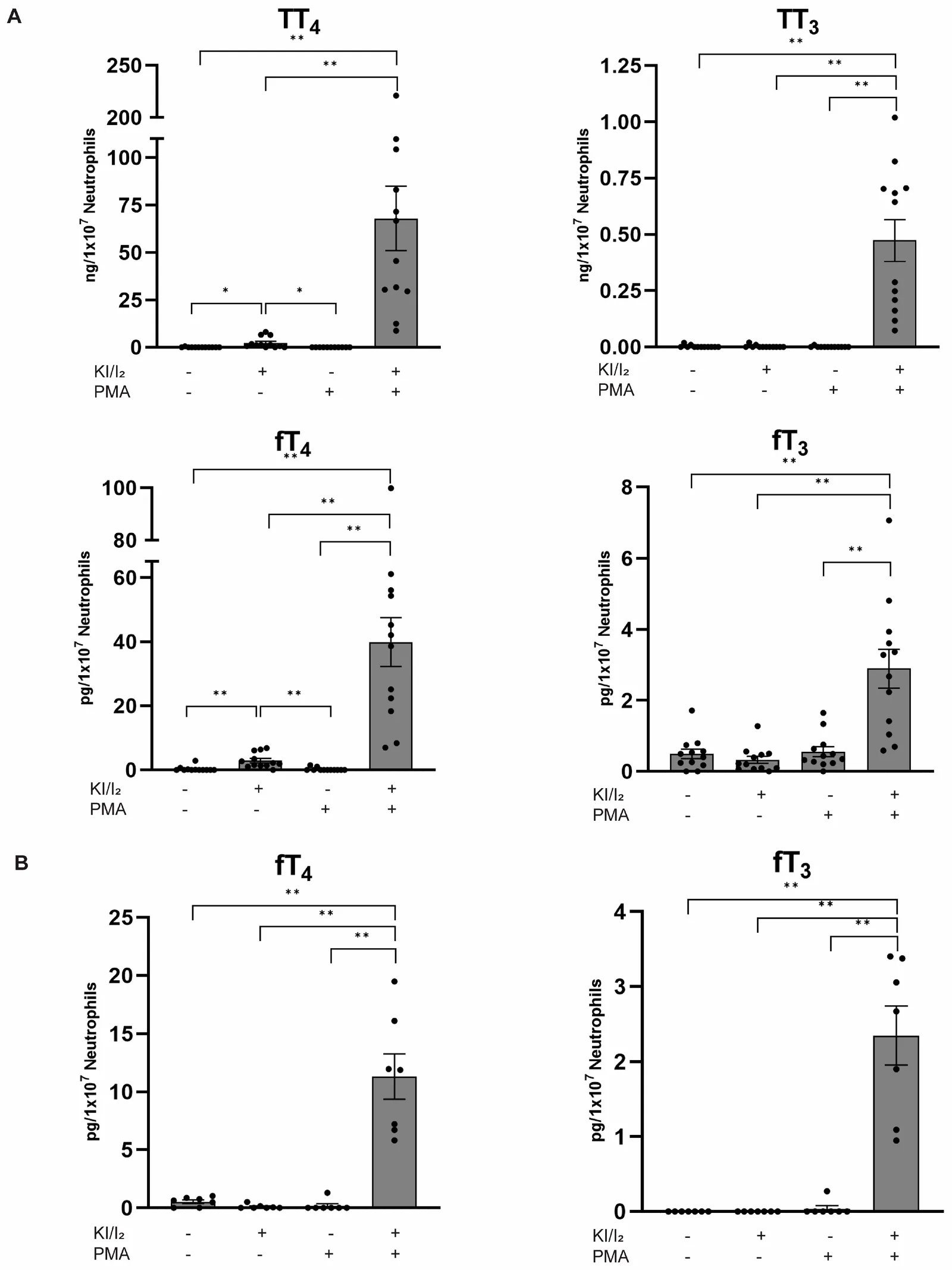
Activated human neutrophils produce thyroid hormone in the presence of iodine. (**A**) Neutrophils were left unstimulated or activated (PMA) in 3% FBS with or without 700 µM of iodine (KI/I_2_) for 48 h. Supernatant was evaluated for free and total T_4_/T_3_ utilizing chemiluminescence immunoassay. Results are presented as mean ± SEM from 11–12 donors. (**B**) Neutrophils were left unstimulated or activated (PMA) in 20% FBS with or without 500 µM of iodine (KI/I_2_) for 24 h. Supernatant was evaluated for free T_4_/T_3_ utilizing chemiluminescence immunoassay. Results are presented as mean ± SEM from seven donors.

Neutrophils produced substantial amounts of T_4_ and T_3_ only in the presence of iodine, when supernatants were probed 48 h post activation (Figure 2A). The results were striking as there was virtually no hormone signal in all other groups, indicating that neutrophil-mediated synthesis required the concomitant presence of iodine and cellular activation. An exception was observed in the group receiving KI/I_2_ without external activation, which showed a small increase in TT_4_/fT_4_ signal above controls (Figure 2A). Of note, our fT_3_ assay is susceptible to interference from biotin levels in media and therefore, although minor, exhibited the highest analytical background signal in longer duration experiments. However, results still showed a strong fT_3_ response in the cells that were both activated and iodine-pulsed (Figure 2A). TT_4_ displayed the highest amounts of hormone, with a mean of 6.02 µg/dL, and one donor producing as much as 20.69 µg/dL of TT_4_. This is in contrast to a below-LOQ mean signal of 0.003 µg/dL in non-activated controls. The group receiving KI/I_2_ without activation signals had a mean TT_4_ of 0.26 µg/dL, which was below LOQ but was reproducible (i.e., statistically significant). The donor-to-donor variability in iodothyronine output may reflect biological variance in expression and activity of thyroid hormone-producing enzymes.

To evaluate the sensitivity of these culture conditions to hormone synthesis, we utilized another group of donors under different experimental conditions, including increased FBS (20%), reduced iodine concentration, and shorter duration of incubation post activation (24 h). In a similar manner, iodine-pulsed activated neutrophils produced thyroid hormone after incubation for 24 h, whereas the other groups had near LOQ T_4_ and T_3_ signals as measured with the more sensitive assays, fT_4_ and fT_3_ (Figure 2B). Utilizing yet another group of donors under the setting of 7% FBS produced the same results (Supplementary Figure S1A). These results show consistent synthesis of thyroid hormones under different conditions, with a detectable hormone signal as early as 24 h post activation.

**Figure 3:**
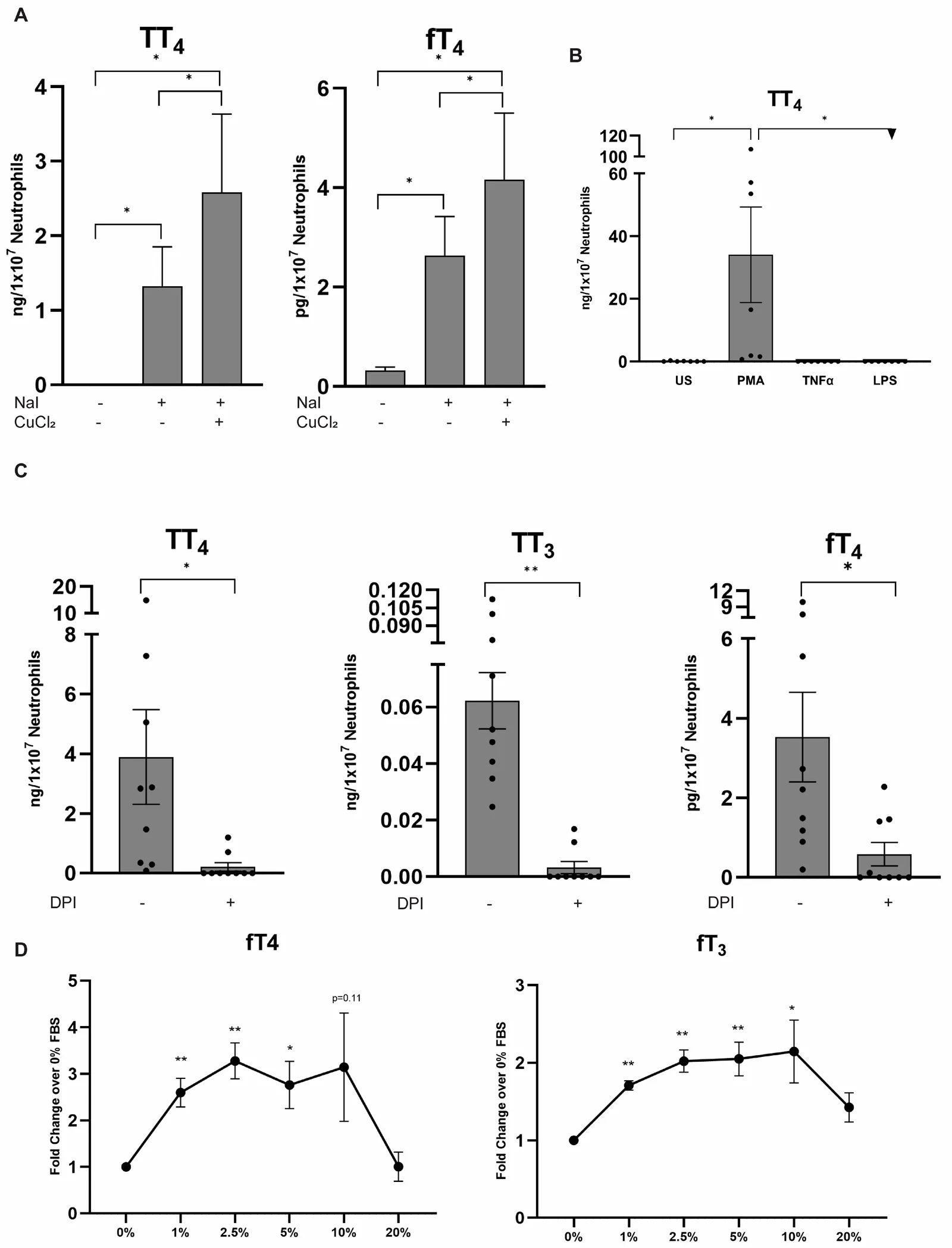
Inhibition of the NADPH oxidase blocks T_4_/T_3_ synthesis by neutrophils. (**A**) Neutrophils were activated (PMA) in 7–10% FBS, 500–1000 µM of iodide (NaI), with or without 3–3.5 µg/mL of copper (from CuCl_2_), as indicated in the figure. The reaction was incubated for 48 h, and the supernatant was evaluated for free and total hormones utilizing chemiluminescence immunoassay. Results are presented as mean ± SEM from 18 donors. Note that this experiment combines two tested conditions (FBS percentage, iodide molarity, and copper concentration) that yield similar results. (**B**) Neutrophils were left unstimulated (US) or activated (PMA, LPS, or TNFα) in presence of 700 µM of iodine (KI/I_2_) for 48 h. Supernatant was evaluated for total T_4_ utilizing chemiluminescence immunoassay. Results are presented as mean ± SEM from seven donors. Arrows in the graph indicate significance to all encompassed groups. Note: the neutrophil “PMA” dataset in Figure 3B is the same dataset shown in Figure 1A, as it was split into two panels for comparison clarity. These different treatments were performed, utilizing same donors within same experimental time frame. (**C**) Neutrophils were activated (PMA) in 3% FBS, 700 µM of iodine (KI/I_2_), with or without 15 µM DPI-DMSO for 48 h. Controls (left bar) received DMSO only. Supernatant was evaluated for free and total hormones utilizing chemiluminescence immunoassay. Results are presented as mean ± SEM from nine donors. (**D**) Neutrophils were activated with PMA in the presence of varying percentages of FBS and 700 µM of iodine (KI/I_2_). The supernatant was analyzed for thyroid hormone fractions using free form for increased sensitivity. Results are presented as mean ± SEM from seven donors.

Next, we utilized a reduced form of iodine [sodium iodide (NaI)] to assess if the oxidation state of iodine would affect thyroid hormone yield. Interestingly, we found that NaI-treated cells had a lower signal for both free and total T_4_. In the case of NaI, some donors had shown undetectable signals, while others exhibited a strong signal above background levels. The addition of copper chloride (CuCl_2_) under these conditions had a modest yet statistically significant increase in hormone yield (Figure 3A). In other experiments, the use of another iodide form (potassium iodide, KI) showed similar effects to NaI. It is plausible that, under our experimental conditions, CuCl_2_ may facilitate the production of more electrophilic species of iodine [2CuCl_2_ + 4NaI → 2CuI + 4NaCl +I_2_], thereby accelerating aromatic iodination of tyrosyl residues. Overall, these results demonstrate that neutrophils can produce T_4_ and T_3_ after loading with KI/I_2_ and, to a lesser degree with reduced forms of iodine (NaI), in conjunction with cellular activation signals.

### 3.3. Appearance of Thyroid Hormone Is Sensitive to Type of Cellular Stimulation

Given the strong reaction to PMA-mediated activation in terms of thyroid hormone production, we asked if other known neutrophil stimulants could produce the same effect. To this end, neutrophils from the same donors were treated either with PMA, LPS, or TNFα. Interestingly, using T_4_ as an iodothyronine probe, we could not detect a signal in the groups receiving LPS or TNFα (Figure 3B). Another experimental setting with higher dose LPS (10 µg/mL) showed the same results; indeed, no T_4_ increase was detected despite combining high-dose LPS treatment with PMA. These results, whilst peculiar, point to potential requirements for synthesis of thyroid hormone that could include extracellular release of both H_2_O_2_ and peroxidases. For example, Mol et al. showed that reactive oxygen species (ROS) production was relatively not effective when using LPS as a stimulant [40]. Therefore, these experiments suggest that the mechanism utilized by PMA-activated neutrophils for forming iodothyronines requires the release of H_2_O_2_.

### 3.4. Synthesis of Thyroid Hormone Is Dependent on the Neutrophil’s Oxidative Burst

Conjugation of iodine onto tyrosyl residues within thyroidal TG requires peroxidase activity in the setting of available H_2_O_2_. The requirement for neutrophil activation via PMA strongly suggested that H_2_O_2_ release mediated by NADPH oxidase is important for iodothyronine formation. To test this, we employed the widely used NADPH oxidase inhibitor, DPI [41,42]. Neutrophils were activated in the presence of iodine, with or without DPI. Remarkably, we observed marked inhibition of thyroid hormone synthesis at 48 h post activation (Figure 3C). These observations demonstrate that inhibition of the NADPH oxidase results in the abolishment of the majority of thyroid hormone produced by neutrophils. In other non-displayed experiments, activation of neutrophils under the setting of lower concentrations of iodine (500 µM) and increased FBS conditions yielded similar DPI inhibition results.

### 3.5. Thyroid Hormone Synthesis by Neutrophils Is Optimal When Serum Components or Thyroglobulin Are Utilized

In our search for optimal conditions for thyroid hormone synthesis, we noticed an inverse parabolic curve of assayed hormones when different concentrations of FBS were used (Figure 3D). Specifically, our immunoassay data showed that neutrophils produce detectable fT_4_ and fT_3_ with and without FBS, but that a concentration range of 1–10% FBS significantly increases detectable levels of hormones in culture media (Figure 3D). Interestingly, these effects are reduced to baseline when the concentration of FBS reaches 20%. Similar observations were shown when total levels of hormones were measured wherein a decrease in TT_3_ production was observed at 20% FBS, yet levels were still higher compared to serum-free media. Based on these results, we postulated the need for proteins with iodination potential, such as TG or albumin, which can be found in high concentrations in normal serum. Compared to albumin, TG is found at lower quantities in plasma but is increased in the setting of hypothyroidism or iodine deficiency [25,26].

Prior to embarking on these studies, we performed a quality assessment on different albumin preparations. We found that undenatured albumin from human serum (>95% purity) is unsuitable for our experimental settings as it displayed high levels of fT_4_ and fT_3_ (>1.5 ng/dL fT_4_, >4.5 pg/mL fT_3_) in a near-physiological preparation of 6.67 g/dL albumin; similar findings were shown for TT_3_ levels. These findings were to be expected, as albumin is a known transport protein for thyroid-derived hormones. To overcome this limitation, we utilized recombinant human albumin, which showed much lower background signals when suspended at 5 g/dL.

**Figure 4:**
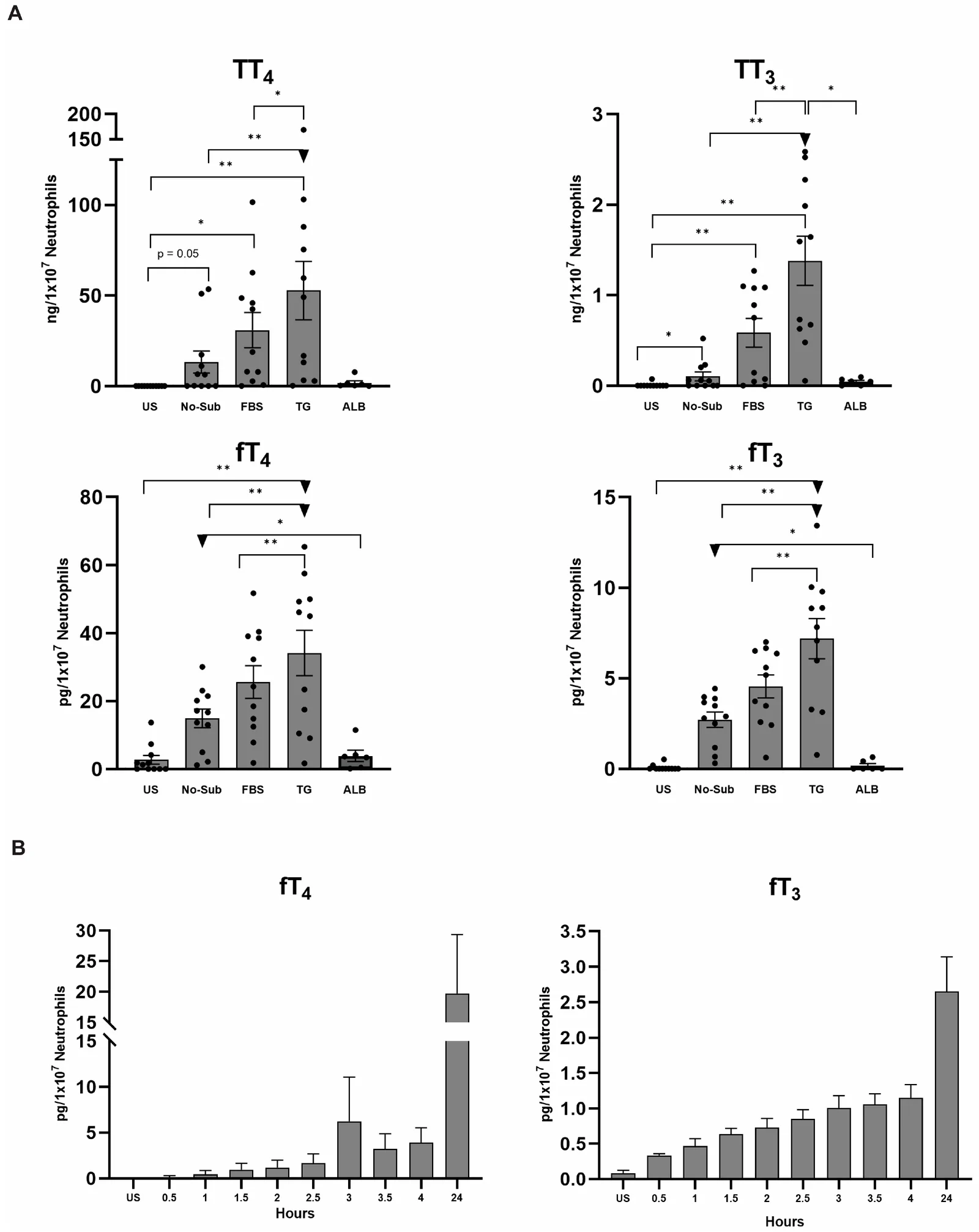
Thyroid hormone synthesis is optimal when TG is utilized as an iodination site. (**A**) Neutrophils were left unstimulated (US—media only) as background control. For evaluating potential protein substrates, neutrophils were activated (PMA) in media only without substrate (No-Sub), and media supplemented with 5% FBS, 20 µg/mL TG, or albumin (ALB) at 5 g/dL. All groups received 700 µM of iodine (KI/I_2_). Cells were cultured for 48 h. Supernatant was evaluated for free and total hormones utilizing chemiluminescence immunoassay. Results are presented as mean ± SEM from 11 donors (6 for ALB). Arrows in the graph indicate significance to all encompassed groups. (**B**) Neutrophils were left unstimulated (US) or activated (PMA, 2.5 µg/mL) in 10% FBS and 20 µg/mL TG, with 700 µM of iodine (KI/I_2_) for varied time points (in hours). Supernatant was evaluated for free T_4_/T_3_ utilizing chemiluminescence immunoassay. Results are presented as mean ± SEM from three donors for all groups except n = 2 for [3, 3.5, and 4 h].

As shown in Figure 4A, after activation of neutrophils with PMA [US (unstimulated) is a non-PMA control] in the presence of no FBS, 5% FBS, TG, or recombinant albumin, we found substantial increases in hormone levels within the FBS and TG fractions (Figure 4A). Perhaps not surprisingly, we found nearly undetectable amounts in the fraction that received recombinant human albumin. Indeed, reactions that contained no exogenous substrate (No-Sub) had higher hormone levels than albumin reactions from the same donors (Figure 4A). Note that all parameters showed statistically significant differences compared to albumin, except for TT_4_ due to donor-to-donor variability. Given that albumin was shown to contain iodination sites for MPO, our results became conflicting since the indication is that albumin may have inhibited MPO’s peroxidase activity. Indeed, previous work showed the potential for albumin to inhibit MPO’s activity [43]. The latter can, in part, explain the inhibition of thyroid hormone appearance at 20% FBS (Figure 3D). Another explanation is that albumin provides tyrosyl iodination sites that do not lead to thyroid hormone production, thereby diverting iodination reactions from iodothyronine pathways.

### 3.6. Production of Thyroid Hormones Begins at Earlier Time Points of Neutrophil Activation

To evaluate earlier time points at which thyroid hormone could be detected, we utilized an experimental setting wherein both TG and FBS are present to maximize hormone yield. Neutrophils were activated, and hormone levels were assessed beginning at 30 min. We observed a gradual increase in the free hormone fractions, with the highest amounts observed at the final time point of 24 h (Figure 4B). Indeed, our experiments shown here at 48 h (Figure 1B and Figure 2A), and 72 h, indicate that hormone levels continue to increase beyond the 24 h time point. Due to the short viability window for neutrophils both in and ex vivo, these observations hinted toward a potential dual mechanism wherein earlier time points allow extracellular MPO and H_2_O_2_ to iodinate TG (or unknown FBS proteins), followed by endocytosis and proteolytic degradation of iodinated proteins for final release of thyroid hormones.

### 3.7. Resting Neutrophils or Protease Activity Liberate Thyroxine from In Vitro MPO/H_2_O_2_-Catalyzed Reactions

To better understand the mechanistic details of iodothyronine synthesis, we formulated a two-part hypothesis based on the following observations. First, our results demonstrate that neutrophil- (or neutrophil components) containing media appears to accumulate thyroid hormone past neutrophils’ expected viability window. Second, the NADPH oxidase inhibitor, DPI, eliminated hormone output presumably due to the reduction of available H_2_O_2_ [44,45]. This is expected as locally synthesized H_2_O_2_ is one of the requirements for thyroid hormone synthesis in the thyroid gland. Based on this, we hypothesized that (A) synthesis of iodothyronines occurs through the extracellular catalytic activity of MPO in the setting of available H_2_O_2_, both of which are released extracellularly upon activation of neutrophils; and (B) similar to the thyroid gland, MPO-iodinated compounds (e.g., TG and FBS) require proteolytic degradation by proteases, which can be derived from neutrophils or other cells, in order to liberate bound thyroid hormone.

**Figure 5:**
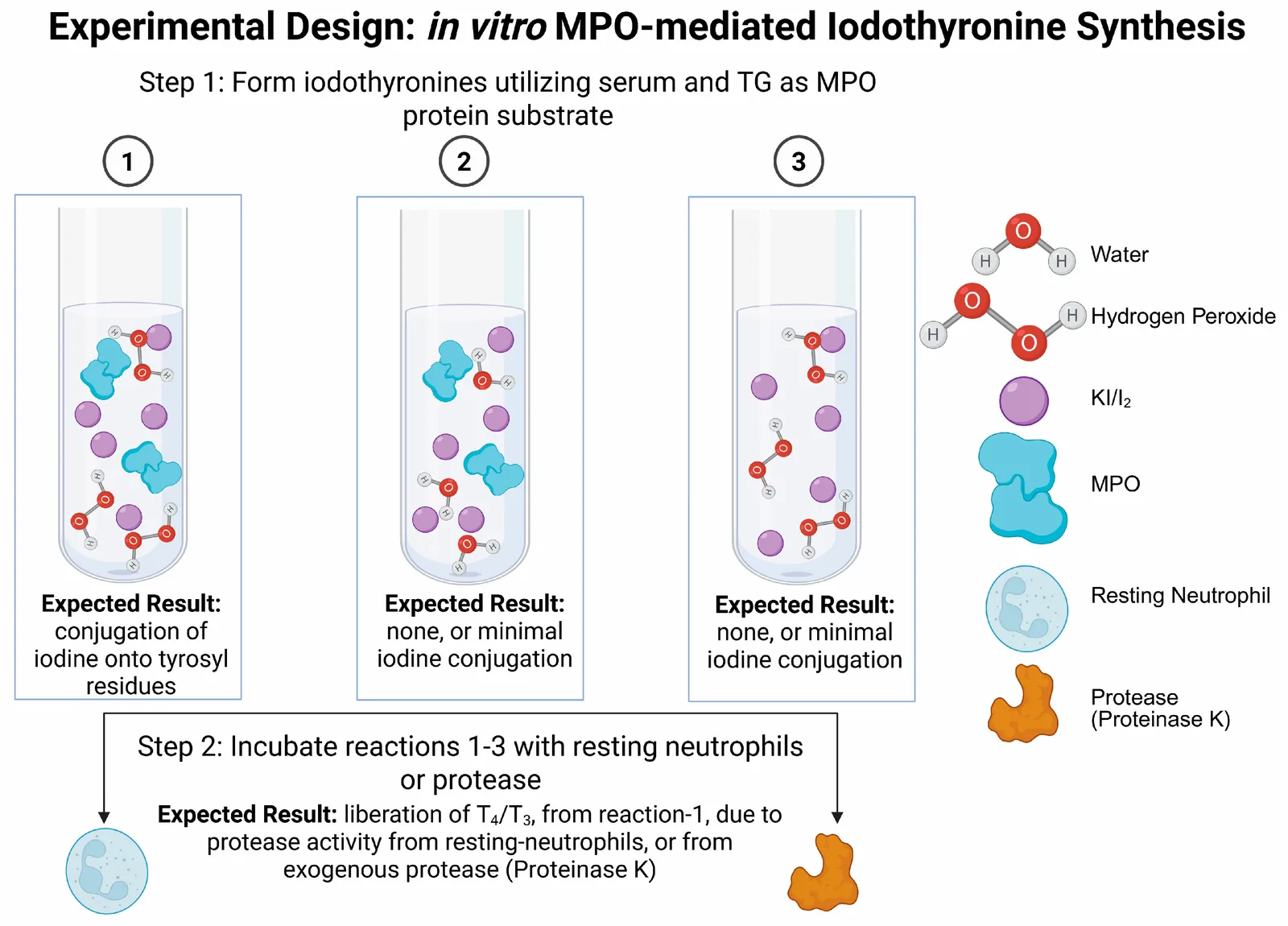
Assay design for MPO-mediated catalysis. Schematic for the three MPO in vitro reactions. Details can be found under the Methods section.

**Figure 6:**
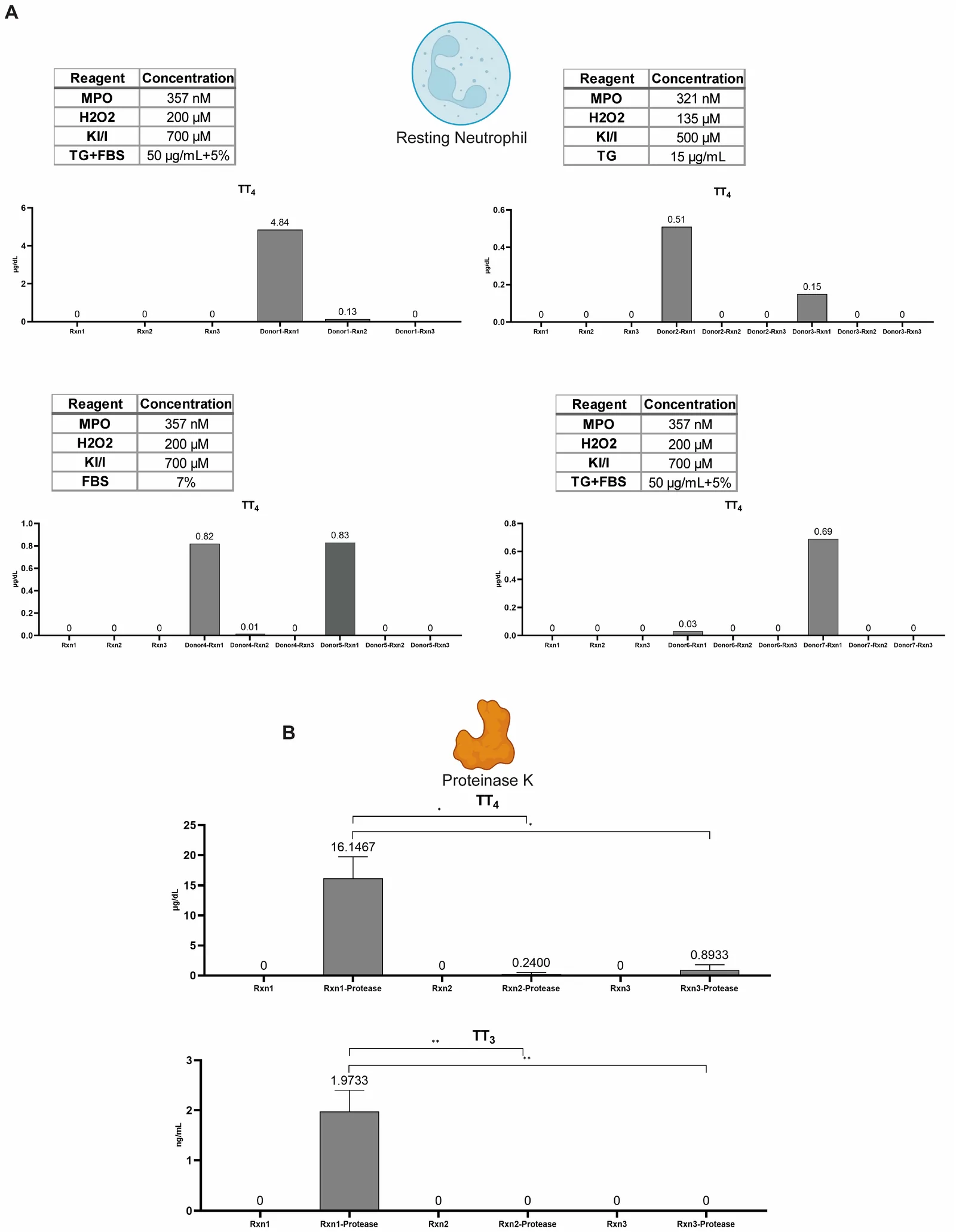
Liberation of thyroid hormone from in vitro MPO-mediated reactions by resting neutrophils and protease. (**A**) Resting neutrophils from seven donors were incubated for 24 h with three in vitro MPO reactions, as described in the Methods section and Figure 5. The conditions used are listed in the table above each graph. Supernatant was evaluated for TT_4_ using chemiluminescence immunoassay. (**B**) The three MPO reactions (see Figure 5) were performed under a single condition, as described in the Methods section. Thermolabile proteinase K was added to initiate proteolytic degradation of TG and iodinated FBS protein matrix. Reactions were collected after inactivation of proteinase K and evaluated for TT_4_/TT_3_ using chemiluminescence immunoassay. Results are presented as mean ± SEM from three independent reactions.

To test this two-part hypothesis, we designed a series of in vitro MPO reactions (reactions 1–3) with an endpoint utilizing either resting (not activated) neutrophils or the broad-spectrum serine protease, proteinase K (Figure 5). The expectation is that only reaction-1 would show the presence of iodothyronines upon treatment with either resting neutrophils or proteinase K. We first used resting neutrophils from seven donors under different conditions, including TG, FBS, or both combined (Figure 6A). As expected, only cells that received the full MPO reaction mix (reaction-1, Figure 5) showed a clear T_4_ signal, albeit the signal was variable based on donors, as observed in the initial activation experiments (Figure 6A). Similar effects were observed when evaluating T_3_. Importantly, reaction-1 did not show T_4_ signal in the absence of resting neutrophils. The latter indicated that there is a probable requirement for proteolytic degradation of iodinated products in order to liberate thyroid hormones. To this end, we utilized one condition for the three in vitro reactions, followed by proteinase K treatment. That is, reactions 1–3 were performed as in Figure 5, and then each reaction was split into two conditions: with or without proteinase K. Remarkably, we detected a substantially higher signal for T_4_ and T_3_ only within reaction-1 (MPO/iodine/H_2_O_2_) that received proteinase K (Figure 6B). Overall, these experiments point toward proteolytic requirements for extrathyroidal iodothyronine liberation.

### 3.8. Resting Neutrophils and Elastase, but Not Cathepsin B, Liberate Thyroxine from Human-Derived Native Thyroglobulin

**Figure 7:**
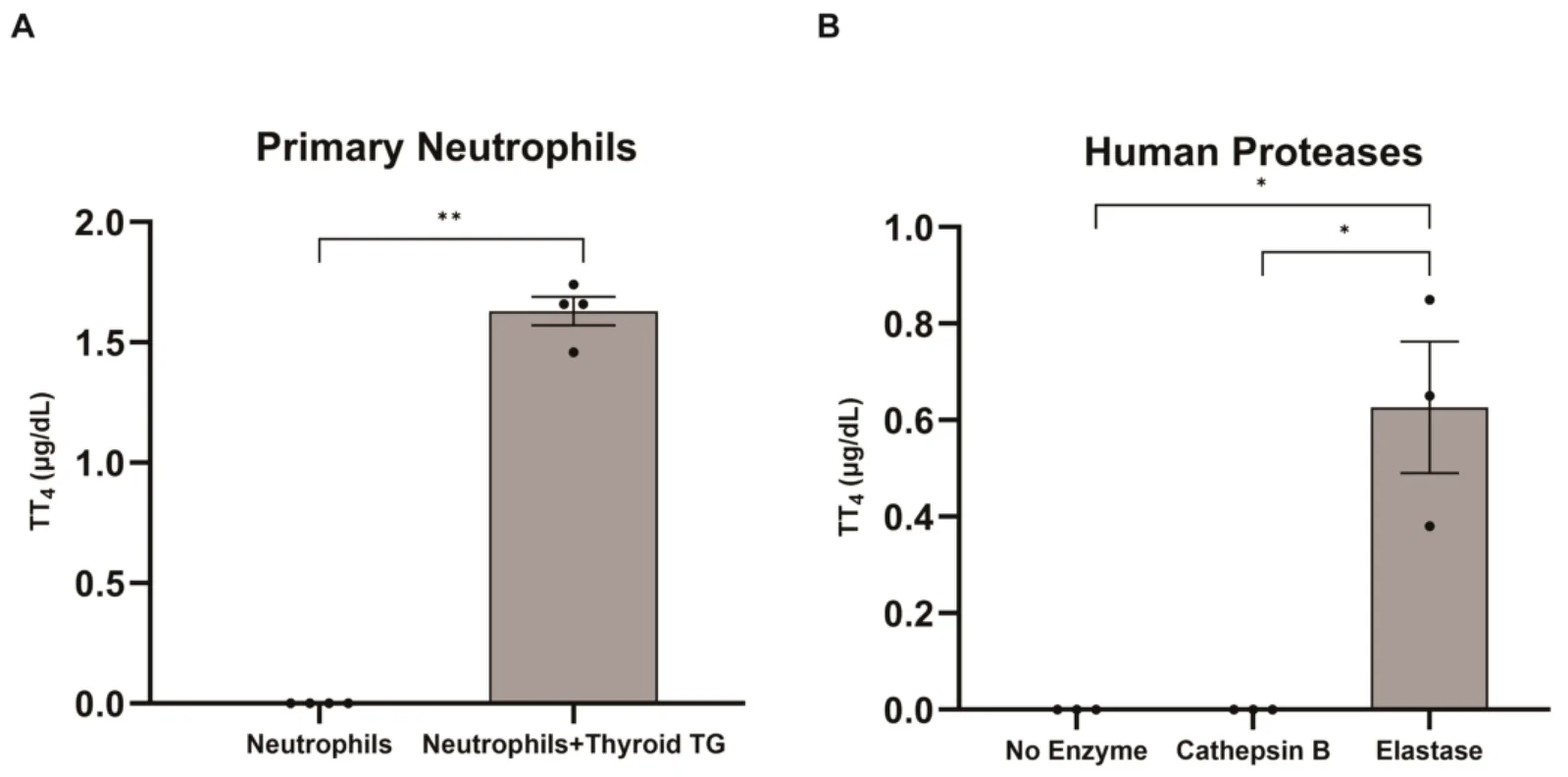
Resting neutrophils and associated elastase liberate thyroid hormone from human-derived native thyroglobulin. (**A**) Resting neutrophils from four donors were incubated for 24 h with or without human thyroid-derived TG (10 µg/mL). Supernatant was evaluated for TT_4_ using chemiluminescence immunoassay. Results are presented as mean ± SEM. (**B**) Neutrophil-derived elastase or liver-derived cathepsin B was incubated with human thyroid-derived TG (10 µg/mL) for 24 h. Reactions were collected and evaluated for TT_4_ using chemiluminescence immunoassay. Results are presented as mean ± SEM from three independent reactions.

Our in vitro enzymatic experiments have shown that MPO-mediated iodination followed by broad-spectrum serine protease, or resting neutrophil, treatment results in release of de novo thyroid hormone (Figure 5 and Figure 6). Based on these results, we asked if pre-iodinated, human thyroid-derived TG could result in release of thyroxine in the same manner. Our 2017 study has already established that PBMC could release thyroid hormone when cells were incubated with human thyroid-derived TG [5]. At the time, it was proposed that this effect was due to proteolytic degradation mediated by immune cells. To confirm this effect in neutrophils, pre-iodinated TG was allowed digestion time by resting neutrophils followed by assessment of total thyroxine levels. As expected, only the neutrophils receiving pre-iodinated TG showed detectable signal of TT_4_ (Figure 7A). Next, we asked if immune-related proteases could mediate these effects, namely, cysteine (cathepsin B) and neutrophil-derived serine (elastase) proteases. Only neutrophil-derived elastase could release thyroxine when incubated with thyroid-derived pre-iodinated TG (Figure 7B). Our results show novel observations wherein the neutrophil-derived serine protease, elastase, directly releases dormant reservoirs of thyroid hormones from naturally iodinated TG derived from human thyroid tissue.

### 3.9. Liquid Chromatography/Mass Spectrometry Confirms Presence of Thyroid Hormone in Activated Neutrophil Fractions

**Figure 8:**
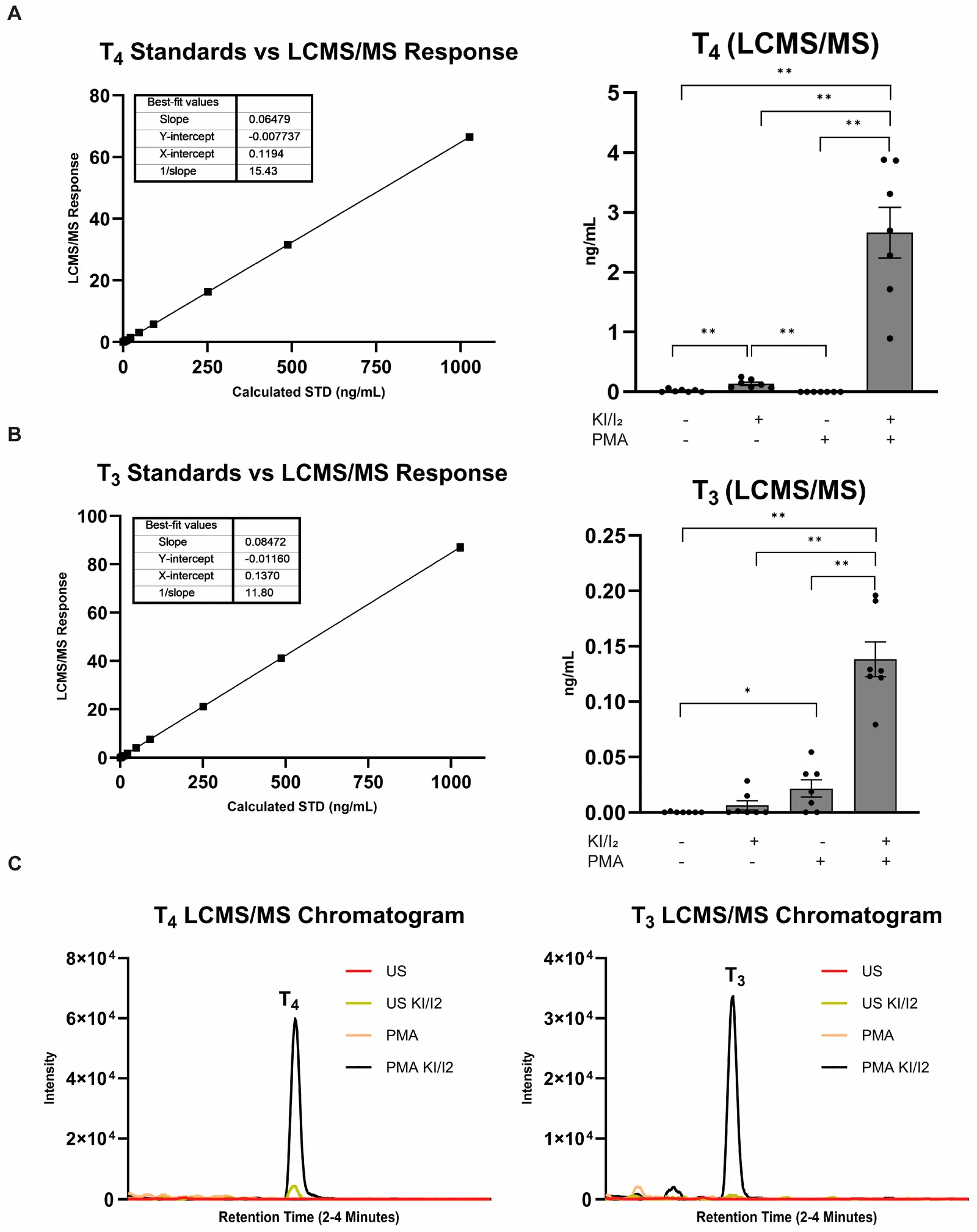
LCMS/MS confirms the presence of de novo thyroid hormone in supernatant from cells receiving iodine and activation signals. Neutrophils were left unstimulated or activated (PMA) in 7% FBS, with or without 700 µM of iodine (KI/I_2_), for 48 h. Supernatant was collected and evaluated for T_4_ (**A**) and T_3_ (**B**) utilizing a direct method [LCMS/MS], as detailed in the Methods section. The left panel shows regression analyses for multiple standards relative to LCMS/MS signals. The right panel shows the detection of thyroid hormones for the four indicated groups. Appropriate blank media was used for each subgroup, and its signal was subtracted from the co-extrapolated LCMS/MS values. Results are presented as mean ± SEM from seven donors. (**C**) Representative LCMS/MS chromatogram from one donor. Shown is retention time from 2 to 4 min from unstimulated (US) or PMA-activated neutrophils, after subtraction of background signal.

Thus far, we have shown results utilizing four IVD-based immunoassays that were rigorously developed in terms of analytical sensitivity and specificity where the assays show non-significant cross-reactivity with similar iodothyronine derivatives. These exact assays are commonly utilized in commercial diagnostic laboratories for the purpose of quantifying human serum-based thyroid hormones. However, given the novelty of this work, it is important to further confirm our observations with a direct method. To this end, we have collaborated with the High-Resolution Mass Spectrometry Facility (University of Iowa) to determine if signals for T_4_ and/or T_3_ could be found within human neutrophil cell culture. A method was developed using a mixture of external and internal (carbon-13) standards, as described in the Methods section and File S1. Regression analysis of standards in relation to LCMS/MS data shows excellent standard to LCMS/MS response, with an R^2^ of 1.00 (Figure 8A,B, left panels). Analytical controls included appropriate blank media for each subgroup, with each blank signal subtracted from co-extrapolated LCMS/MS values. Overall, all groups had comparable blank signals, indicating no analytical interference from iodine or PMA.

Analysis of the four treatment groups with seven donors showed similar output compared to our immunoassay analysis (Figure 8A–C). Neutrophils receiving both iodine and activation signals showed substantial T_4_ and T_3_ signals, whilst the other groups had minimal or undetectable levels of these hormones. Of note, similar to immunoassay results, groups receiving only iodine showed a small but statistically significant signal for T_4_. LCMS/MS detection of thyroid hormones confirms our immunoassay findings and demonstrates the appearance of de novo thyroid hormone after activation of iodine-loaded neutrophils.

## 4. Discussion

**Figure 9:**
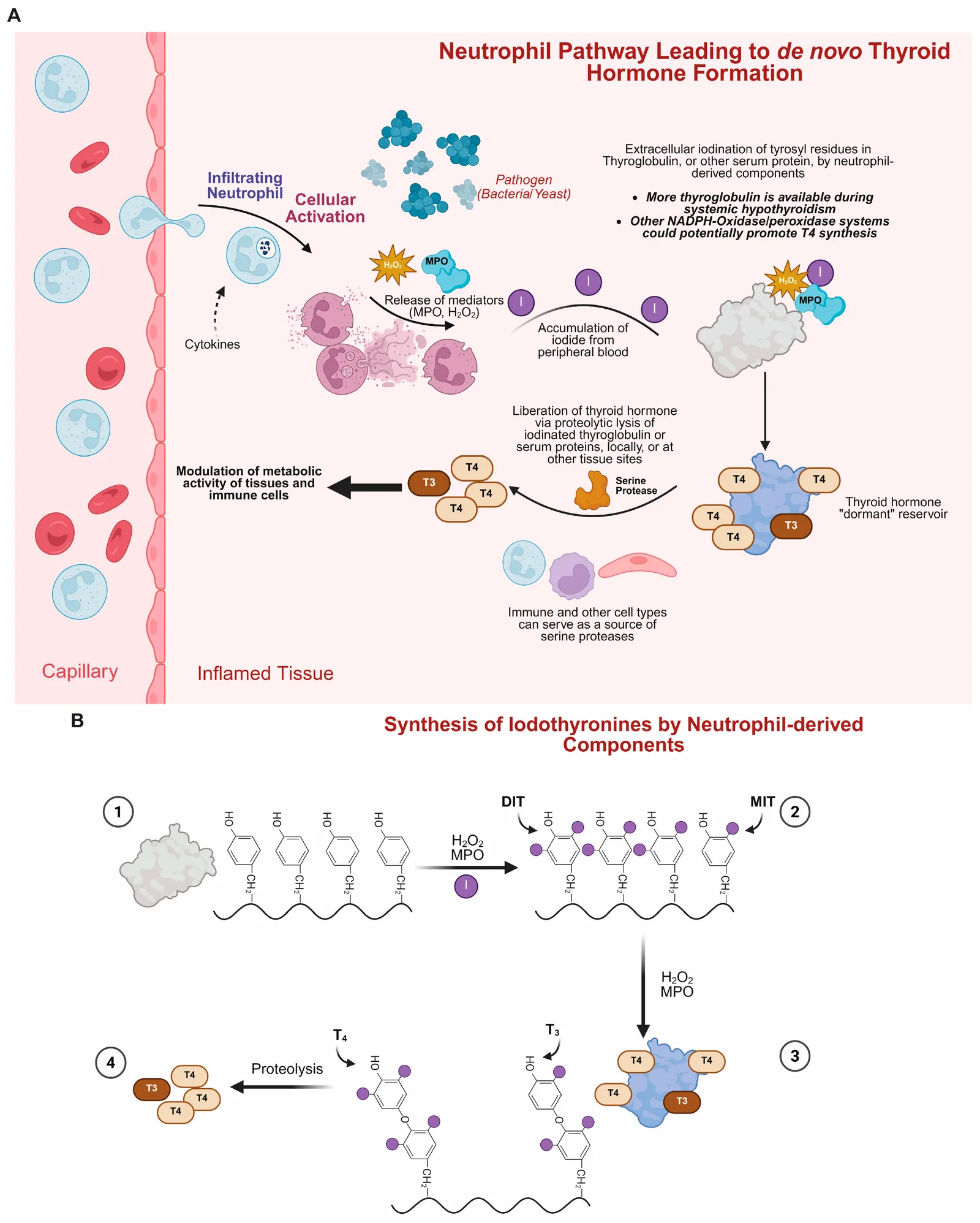
Proposed model—extrathyroidal synthesis of de novo thyroid hormones. (**A**) Neutrophils are recruited to infection or inflammatory sites wherein cellular activation occurs. This leads to a host of neutrophil-mediated effects, including the release of elastase, MPO, and the production of H_2_O_2_. Available iodide, along with iodination sites (plasma proteins and/or TG), promotes formation of dormant (unreleased) reservoir of thyroid hormone. Proteolytic degradation of hormone reservoir occurs through action of serine proteases found in the local protein milieu and/or proteases derived from various cell types (e.g., monocytes and endothelial cells). The release of thyroid hormones could potentially promote metabolic and immune cell activity around infection sites. Alternatively, during systemic hypothyroidism, TG is known to increase in plasma. In this scenario, the increased TG could act as an MPO substrate, thereby providing a larger reservoir of thyroid hormones, given the availability of iodine species along with alternative H_2_O_2_-generating systems (NOX/DUOX NADPH oxidases). NADPH oxidases may be localized in immune, hepatic, intestinal, and mammary tissues. Overall, thyroid hormone regulation is a dynamic process regulated at several key steps that include regulation by thyroid-stimulating hormone (TSH) and DIO enzymes, which can convert T_4_ → T_3_. Given this, we propose that the mechanisms delineated here serve as a downstream fine-tuning mechanism to concentrate thyroid hormones within tissue sites. (**B**) Biochemical representation of iodination and coupling reactions on the backbone of TG or other substrate proteins. (1) Tyrosyl residues on the peptide backbone of TG or other substrate proteins; (2) conjugation of tyrosyl residues with reactive oxidized iodine through the action of MPO/H_2_O_2_, thereby forming MIT/DIT residues; (3) MPO promoting intramolecular coupling of MIT/DIT to form T_4_ and T_3_ hormones on iodination sites; and (4) proteolysis resulting in release of T_4_ and T_3_.

Elucidating if and how de novo thyroid hormones are produced, and the extent to which their interaction affects localized tissue-level iodothyronines, would be vital to our understanding of immunity, reproduction, and cellular metabolism. In this work, we have significantly expanded on the century-long exploration for potential extrathyroidal sources of thyroid hormones. We hypothesized that the neutrophil’s oxidative burst and MPO activity would result in the organification of iodine into thyroid hormones by utilizing TG, or serum-derived substrates. Our observations show that ex vivo activation of human neutrophils results in appearance of substantial amounts of T_4_ and T_3_ when either serum or TG was used as protein iodination sites. It is unclear if more than one serum protein substrate is involved in iodothyronine synthesis. We could not detect the presence of thyroid hormones without iodine or induction of the oxidative burst, as shown by DPI inhibition experiments. Activation of iodine-pulsed neutrophils with LPS or TNFα did not produce detectable thyroid hormones, presumably due to lower release of ROS and/or MPO. A different form of iodide (NaI) displayed similar effects, yet with a lower yield. The addition of CuCl_2_ to the NaI reaction mix resulted in an enhanced T_4_ signal, which suggests that the oxidative state of iodine is important for the propagation of tyrosine iodination. Mechanistic experiments demonstrated that both MPO and H_2_O_2_ are necessary for producing thyroid hormones. Interestingly, T_4_ and T_3_ were liberated from iodinated proteins only when the MPO/H_2_O_2_ reactions were incubated with non-activated (resting) neutrophils. We hypothesized that the effects of liberation were attributable to protease activity. Indeed, treatment of the in vitro MPO/H_2_O_2_ reactions with serine protease, proteinase K, liberated high levels of thyroid hormones, with undetectable signals in the group not receiving proteinase K. Reactions without MPO or H_2_O_2_ did not show a thyroid hormone signal regardless of protease or neutrophil treatment. Similarly, neutrophil-derived serine protease (elastase), but not the cysteine protease (cathepsin B), released T_4_ from thyroid-derived thyroglobulin. Indeed, proteolytic liberation of thyroid hormones from iodinated targets (e.g., TG) is a known mechanism in the thyroid gland and has been previously shown to occur in human leukocytes by our group [5]. Our data suggest a sequential mechanism that includes NADPH oxidase, MPO, and serine protease activity for the liberation of iodinated proteins. Therefore, non-thyroidal cells have the potential to synthesize de novo hormones, which may contribute to changes in localized iodothyronine concentrations under specific inflammatory conditions (Figure 9).

Optimal thyroid hormone formation occurred in the setting of either TG or FBS. This is not surprising, as peroxidase-mediated iodination has been shown to occur in multiple proteins, such as TG, casein, and serum globulins [18,20,28,46]. We expected that albumin would be a suitable iodination/coupling site for hormone production, but in contrast to mid-twentieth-century studies, we found that our experimental conditions could not generate thyroid hormone signals in the presence of recombinant human albumin [18,23]. It may be that the use of recombinant albumin may have reduced the affinity of iodide for MPO [43]. Unfortunately, alternative plasma-derived undenatured preparations of human albumin harbor a significant amount of thyroid hormone and therefore cannot be used in our experiments. Future work should detail the mechanisms and kinetics of TG iodination and further explore the identity of other proteins with iodination potential. This work should also define optimal conditions in terms of minerals or vitamins that enhance this response. For example, we have shown here that CuCl_2_ could enhance NaI-mediated iodothyronine synthesis. Table 2 summarizes parameters that were essential, or had optimized, the resultant thyroid hormone release.

It is essential to note that, in most experiments, we have used relatively long incubation times post-neutrophil activation (24 and 48 h). We shall mention here that assuming that the hormone production observed here is not possible only because neutrophils have a short activation/life cycle is not valid evidence against this work. The authors are aware that neutrophils inherently have a short viability period, as well as short activation and shutdown time points—specifically for NADPH oxidases (in minutes). The reasons for commencing on these longer time points are twofold. First, not all neutrophils become apoptotic in the first hours post activation, as independent investigators show close to 40% survival at 48 h, with live neutrophils observed even at 72 h [47,48,49,50]. In particular, the recent work of Kolman et al. demonstrated these points in terms of neutrophil survival, activation parameters, and, importantly, MPO activity at 48 h post PMA activation [47]. In our hands, long stimulatory conditions with high-dose PMA indeed resulted in cell death of almost all neutrophils. However, even if neutrophils do not survive shortly after activation, we would still be interested in the enzymatic activity of MPO and proteases post cell death, particularly as MPO’s extracellular coupling activity is expected to continue beyond the short NADPH oxidase activation timing. Thus, our goal was to maximize the resultant biochemistry post activation (i.e., number of neutrophils and released bio-machinery), as well as extend the time points to observe maximal potential hormone production. Indeed, time point studies (Figure 4B) show evidence for a continuous process post NADPH oxidase activation—akin to ongoing cytokine release (hours/days) post T-cell receptor activation or calcium influx, which both occur and diminish in seconds to minutes [39]. Indeed, our observations pointed toward a multi-step process that begins with NADPH oxidase activation, releases MPO, and ends in protease-mediated liberation of dormant hormone reservoirs (i.e., iodinated proteins).

One of the interesting observations encountered during our study is the apparent inhibition of hormone production at higher levels of FBS and the presence of an inverse parabolic curve of hormone release (Figure 3D). While peculiar, this observation hinted toward the mechanism in place, that is, MPO-mediated catalysis in the presence of H_2_O_2_. It is highly probable that the inhibition occurred due to the increasing concentrations of MPO- and ROS-inhibiting components. First, the copper-containing plasma protein ceruloplasmin is known to bind MPO with high affinity, thereby causing inhibition of peroxidase activity [31,51,52]. Second, serum proteins such as glutathione peroxidase can reduce the availability of H_2_O_2_ produced by neutrophils [31,53]. Third, as our results suggest, different forms of albumin could potentially function as inhibitors of extrathyroidal hormone synthesis [43]. We have also found that the NADPH oxidase inhibitor (DPI) carrier solvent (DMSO) reduced total levels of hormones in the media. Beilke et al. showed that DMSO suppressed the production of superoxide, H_2_O_2_, and hypochlorous acid in human neutrophils [44]. Others show similar effects of DMSO on neutrophil activity [45]. These effects are in part due to ROS scavenger properties of DMSO [54]. Thus, the reduced hormone output in higher concentration FBS, or DMSO-treated neutrophils, pointed toward an important component of the mechanism for iodothyronine synthesis (i.e., H_2_O_2_).

Next, for in vitro MPO iodination, we utilized two different molar ratios of H_2_O_2_:MPO (approximately 420:1 and 560:1). Paumann-Page et al. showed that high molar ratios (>1000:1) can inhibit MPO in a manner dependent on the concentration of H_2_O_2_. For example, there was approximately 40% and >90% inhibition of MPO at 1000:1 and 10,000:1 ratios, respectively, when assessed at 60 min of reaction time. The authors show that the loss of MPO activity was independent of the delivery method for H_2_O_2_ (e.g., a one-time bolus of H_2_O_2_ vs. a gradual H_2_O_2_ pulse over 60 min). Based on this, we utilized an H_2_O_2_:MPO molar ratio below the 1000:1 range [55].

The reader may understandably be concerned regarding the assertion of de novo hormone synthesis. In particular, could the detected T_4_ be a result of interference from hormones already present in serum and/or pre-iodinated protein substrates? This was a crucial point to address at the beginning of our study, and as such, we have rigorously performed quality checks to demonstrate that any hormone detected is a result of new biosynthesis. First, we have tested all culture media/serum with four IVD-based immunoassays as well as LCMS/MS (performed by another institution). The result is that no detectable thyroid hormone was found in media/serum, as clearly shown by our varied experiments. These immunoassays are utilized for diagnostic purposes, and we refer the reader to the Methods section for details on assay interference and sensitivity. Second, we have avoided introducing hormone carriers (e.g., see Results section regarding human-derived albumin) to culture media. We have also excluded the presence of iodinated substrates or hormone-carrying proteins in FBS, as shown by both ex vivo and controlled enzymatic experiments. For example, proteinase K and elastase show no detectable signal of T_4_/T_3_ when incubated within cell media/serum. In fact, further demonstrating our proposed mechanism, appearance of hormones only occurred when iodinated substrates were added (i.e., TG iodinated in vitro or obtained naturally iodinated from human thyroid tissue). Indeed, if there were significant interfering amounts of iodinated substrates in the serum, we would have observed substantial release of T_4_/T_3_, but this was never encountered. Given the above, we cannot attribute the appearance of T_4_/T_3_, which is sensitive to conditions detailed in our study, other than to de novo synthesis by neutrophil bio-machinery.

Regarding mechanistic sequences, we propose that there are two cellular mechanisms at play. First, post-neutrophil activation, MPO-mediated iodination of specific proteins occurs concomitantly with the coupling of MIT/DIT by MPO. These are likely maximal within the first minutes to the early single hours, depending on the system, supply, and concentrations of proteins such as TG, as well as inhibitory plasma- and tissue-derived compounds. The kinetics of this response can potentially be extended if there is a continuous supply of H_2_O_2_ (either in vivo or ex vivo through a glucose–oxidase system) that is not inhibitory to MPO [55]. To this end, in vitro iodination studies utilizing TPO showed that formation of T_4_/MIT/DIT on TG occurs at short intervals (maximal at 20 min) relative to in vitro neutrophil survival time [56]. Second, and importantly, we propose that iodinated proteins carrying coupled MIT/DIT, or T_4_/T_3_, are in a “dormant” state until exposed to proteolytic systems (Figure 9). Indeed, proteolytic lysis of hormone reservoirs (e.g., iodinated TG) occurs in the thyroid in part through the action of cathepsins [12]. Therefore, we expect that formed T_4_/T_3_ stay dormant until exposed to a proteolytic system, regardless of whether or not proteins are maximally iodinated in seconds or hours.

An important question to address is how these ex vivo experiments relate to physiological scenarios in which de novo extrathyroidal hormone is produced. Indeed, it has been observed that thyroid hormone concentrates substantially at sites of infection, such as pulmonary abscesses [57]. Thyroid hormones were found to concentrate in leukocytes actively phagocytizing bacteria. Others found that bacterial meningitis alters thyroid hormone profile at infection sites (CSF) in conjunction with neutrophil influx [58]. By the authors’ calculations, at least 15% of the serum thyroxine would be required to leak into CSF compartments to explain the substantial increases of thyroxine [58]. In light of new evidence in this work, we propose that at least some of the thyroid hormone found in CSF during an infection could be a result of de novo mechanisms, akin to what is demonstrated within our ex vivo systems. However, these observations must also be considered in terms of non-thyroidal illness syndrome (NTIS) in patients who are critically ill or are diagnosed with sepsis. In these cases, systemic serum concentrations of T_4_/T_3_ drop substantially, concomitant with an increase of rT_3_ (inactivated T_3_) [58,59,60]. For example, in one study utilizing critically ill patients, mean serum levels of T_4_ were shown to be approximately 3.49 µg/dL (45 nmol/L), wherein the typical normal range from our clinical laboratory is 6.09–12.23 µg/dL [61]. Another study demonstrated a similar decrease, wherein the sepsis group showed a mean T_4_ at 4.7 µg/dL, compared to the control group presenting with a mean of 8.4 µg/dL [62]. The NTIS mechanism of reduced systemic thyroid hormones is not fully understood, but it is known to involve reduced binding of T_4_ to thyroid hormone carrier proteins, such as thyroxine-binding globulin or albumin [60,62]. Another important factor to consider is peripheral deiodinase activity, which can be regulated by cytokines. In addition, since neutrophils express high levels of DIO type 3, under certain conditions, the loss of T_4_/T_3_ from periphery could be attributed to enzymatic conversion of T_4_ to rT_3_ [58]. The latter is expected to be complicated by the dynamic differential expression of different DIO under different conditions. Given this, we rather predict that the effects of thyroid hormone regulation by neutrophils (via DIO or de novo synthesis) is localized within contained infection/inflammatory sites, such as CSF meningitis, and that no significant systemic effects are predicted. That is, our findings here should not be interpreted as predicting increased circulating thyroid hormone concentrations during sepsis and that local production within inflammatory microenvironments may coexist with systemic hormone reduction in NTIS or in critically ill patients. Next, to be complete in our assessment, we must relate that thyroidectomized rats were found to contain unexpected levels of serum T_4_ when given iodide under non-inflammatory conditions [13,14]. Therefore, the mechanisms we propose must be defined in future studies under different contexts (i.e., inflammatory, infectious, and normal conditions).

One limitation in this study is that we have utilized a widely used, but non-physiological stimulus, PMA. Whilst the core purpose of this work is to provide a proof-of-concept cellular study for extrathyroidal hormone synthesis, it would be important to follow this work with confirmatory ex or in vivo studies. Future work should highlight physiological pathways that lead to thyroid hormone production. We shall note here that in our preliminary experiments, PMA stimulation at lower doses produced a similar response, albeit with a lower output of thyroid hormone. On the other hand, we show here that relatively high doses of LPS or TNFα did not produce measurable thyroid hormone. This is despite the observation that neutrophils stimulated with TNFα are capable of producing ROS [40]. Indeed, some of the differences observed between stimulants are due to differences in concentration for released ROS [40]. Degranulation and concentration differences for released MPO and proteases likely contribute to the observed differences between stimulants. This stimulant effect can be observed in the process of NETosis. For example, while NETosis is known to occur in vivo, some studies show that under specific experimental conditions, PMA, but not LPS or TNFα, could induce this effect in vitro [40]. Thus, we speculate here that some of the mechanisms leading to NETosis may share similar pathways to neutrophil-mediated thyroid hormone synthesis, such as the release of MPO and other enzymes [63,64,65].

Given this, we propose that, in vivo, the first batch of neutrophils that are activated (e.g., during infection) can mediate iodination of plasma proteins through the action of H_2_O_2_ and MPO. Next, additional reinforcement of neutrophils or other innate cells arrive at the local iodination site and proteolytically degrade iodinated sites, followed by release of thyroid hormones (Figure 9). Other tissues capable of synthesizing serine proteases (e.g., hepatic cells) would most likely be able to liberate thyroid hormones from peroxidase-mediated iodination. It is important to note here that, in addition to TG, plasma MPO levels have been shown to increase in at least one study during systemic hypothyroidism [66]. Whether or not the increase in TG or MPO is an attempted physiological response to raise levels of thyroid hormone remains unclear.

Neutrophils also play an important role in the context of reproductive immunology and infertility, as these cells become relatively abundant in the endometrium concomitant with progesterone decline [67,68]. Indeed, neutrophils constitute a significant fraction of total endometrial cells estimated at 6–15% of the total endometrial cell number [69]. The role of neutrophils in the endometrium has been suggested to include tissue removal and repair during the normal menstrual cycle [70]. In endometriosis, infiltrating immune cells such as neutrophils contribute to inflammation through cytokine and chemokine secretion [71,72,73]. We therefore propose that the dynamics of thyroid hormone within localized reproductive tissue could, in part, be controlled by infiltrating neutrophils. The latter would involve both DIO activity by neutrophils (i.e., transition from T_4_ → T_3_), but also synthesis and/or liberation of de novo hormone, as we have demonstrated in this work.

We shall mention that the ideas presented here are not new in terms of compensatory or redundant mechanisms utilized by different tissues. For example, synthesis of cholesterol-derived steroids occurs in multiple sites that include the adrenal gland, ovary, placenta, and adipose tissue [74]. TSH release is not limited to the pituitary gland and indeed was found to be produced by CD45 + CD11b+ and small intestinal epithelial cells [75,76]. In the case of thyroid hormones, the thyroid gland is considered the main site of thyroxine synthesis, with the status quo of continuous thyroid hormone release. Given the expression of thyroid hormone-producing bio-machinery in bodily tissues, it should not be surprising to observe that, under specific contexts, other cell types could display thyroid hormone-producing capacity. We show this here in the context of activated neutrophils, but other sites could likely perform the same function. For example, ovarian concentration of iodide has been shown to be second to the thyroid [77]. Monteleone et al. discussed that thyroid hormones are detected in ovarian follicular fluid in concentrations higher than those in serum [78]. The latter is a similar observation to the high concentrations of T_4_/T_3_ found in the CSF in patients with bacterial meningitis [58]. Monteleone et al. showed the presence of TPO by immunocytochemistry and also suggested the presence of extrathyroidal thyroid hormone-producing machinery within the ovary [78]. Another probable site for extrathyroidal hormone synthesis would be mammary cells that can secrete the iodination-capable enzyme LPO and have concomitant expression of NADPH oxidase, NOX4 [79,80,81,82]. To this end, casein protein was shown to be iodinated, resulting in detectable T_4_ [20]. Similarly, stomach cells and subpopulations of hepatic (Kupffer, Stellate) cells express peroxidases and/or NADPH oxidases [37,83,84,85]. Gamma globulins also showed a high potential for in vitro iodination utilizing LPO and H_2_O_2_ [86]. Thus, given the plasticity of the human organism, in terms of protein and enzyme redundancy, along with multiple independent experimental evidence, it should not be surprising that thyroid hormones can be produced in non-classical systems—to a lesser extent and under certain biological contexts.

## 5. Conclusions

In conclusion, our ex vivo experimentation using the human neutrophil demonstrates that thyroid hormones could indeed be produced outside of the thyroid gland under specific stimulatory conditions. Our work further strengthens decades of suggestive hypotheses derived from fragmented in vitro experiments, as detailed herein. Indeed, this highlights another role for extrathyroidal iodine, which has recently been explored in the context of immunity, fertility, and promising cancer treatments. We have also attempted to define some of the molecular mechanisms that could lead to extrathyroidal hormone synthesis in vivo. Our data point to a mechanism of extrathyroidal thyroid hormone synthesis that is likely compartmentalized into two phases: initial iodination (minutes to hours) to increase “dormant” hormone in terms of iodo-protein reservoirs, followed by proteolytic degradation (hours to days) for final hormone release. The latter is likely mediated by proteases found in multiple cell types that include immune and hepatic cells. Future work should define the kinetics of hormone production, optimal ratios for MPO-mediated iodination, the role of copper compounds, the identity of other iodinated plasma proteins, in vivo infection contexts, and evaluation of alternative proteases that could liberate thyroid hormones from dormant iodinated proteins.

## Figures and Tables

**Table 1:** Comparison of protein expression and biochemical output between neutrophils and the thyroid gland.

Substance/Protein	Thyroid Gland	Neutrophils
Iodide Transporters	NIS/PENDRIN	NIS/PENDRIN
NADPH Oxidase	NADPH oxidase dual oxidase (DUOX)	Phagocyte NADPH oxidase (NOX2)
H_2_O_2_ Production	Capable—required for thyroid hormone synthesis	Capable—post activation
Peroxidase	TPO	MPO
Oxidation of Halides	Oxidation of iodide → iodine as a function of TPO activity in the presence of H_2_O_2_	Hypohalous acids from halogens such as chloride and iodide (i.e., iodide oxidation). Occurs as a function of MPO activity in the presence of H_2_O_2_
Abundance	Can concentrate essential components (e.g., TG and iodine) in a localized region (thyroid colloid/lumen)	Abundant immune cell can traffic and concentrate/activate in localized regions, such as inflammation sites or CSF that may contain plasma/tissue-derived iodination sites (e.g., TG)

**Table 2:** Effects of substances on neutrophil-mediated thyroid hormone production.

Parameter	Effect
Core Substances: KI/I_2_ & PMA	↑↑↑↑↑
NaI/KI	↑
NaI/KI + Cu	↑↑
3–10% FBS ζ	↑↑↑↑↑
>20% FBS ζ	↑/=
TG ζ	↑↑↑↑↑
Albumin ζ	↓↓
Duration of incubation	↑ directly proportional

ζ compared to no serum. ↑ or ↓ indicate varied intensities for increase or decrease, respectively.

## Data Availability

The original contributions presented in this study are included in the article/Supplementary Materials. Further inquiries can be directed to the corresponding author.

## Supplementary Materials

The following supporting information can be downloaded at: https://www.mdpi.com/article/10.3390/biom16081081/s1, Figure S1: Human neutrophils produce thyroid hormone after activation in the presence of iodine; (A) Neutrophils were activated with (PMA, 2.5 µg/mL) in 7% FBS with or without 700 µM of iodine (KI/I2) for 48 h. Supernatant was collected and evaluated for free and total T4/T3 using chemiluminescence immunoassay. Results are graphed ± SEM of six donors. Note that one iodine-treated donor had a fT4 signal above the linear range of the instrument (>6.15 ng/dL). In the quantification and graph, the value for this donor was set at fT4 = 6.15 ng/dL; File S1: Protocol for Thyroxines Measurement by UI HRMSF.
