# Supplementary material for: Human Neutrophils Produce De Novo Thyroid Hormones Mediated by the Induction of the Oxidative Burst
Source: Biomolecules. 2026 Jul 23;16(8):1081. doi: 10.3390/biom16081081 (PMC13511619; doi:10.3390/biom16081081)
Supplement: Supplementary file 1 [file biomolecules-16-01081-s001.zip › File S1.pdf]

Protocol for Thyroxines Measurement by UI HRMSF

MATERIALS:

Primary Standards

Primary standards	Manufacturer	Part Number
L-Thyroxine (T4)	Sigma	T-073
3’,3’,5’-Triiodo-L-thyronine (T3)	Sigma	T-074

Internal Standards

Internal standards	Manufacturer	Part Number
L-Thyroxine-13C6 (T4-13C6)	Cayman	T-076
3’,3’,5’-Triiodo-L-thyronine-13C6 (T3-13C6)	Cayman	T-077

Chemicals

Reagents	Manufacturer	Part Number
Methanol (Optima LC/MS grade)	Fisher LC/MS optima	A456
Acetonitrile (Optima LC/MS grade)	Fisher LC/MS optima	A955
Formic acid	ThermoFisher Scientific	28905
Water (Optima LC/MS grade)	Fisher LC/MS optima	W6

Equipment

Columns/Instruments/ Tubes/ Vials	Manufacturer	Part Number
Acquity BEH C18 (2.1 X 100 mm, 1.7 µm)	Waters	186009455
300ul Autosampler vials	ThermoFisher Scientific	6PSV9-03FIVP
Autosampler caps, 9mm	Thermo Fisher Scientific	6PSC9ST101
LP Vortex mixer Tube holder (10mm)	Thermo Fisher Scientific	
Sorvall Legend Micro 21R Centrifuge	Thermo Fisher Scientific	
Labconco RapidVap Vertex N2 Evaporator	Thermo Fisher Scientific	

PROCEDURE:

- Standard Stock Solutions:
  - Primary Standard Stock -100 ug/ml in Methanol with 0.1N NH3.
  - Internal Standard Stock – 13C-labeled standards at 100 ug/ml in Methanol with 0.1N NH3
- Standard Stock Dilutions:
  - Internal Standards (ISTD) were mixed in 5 ml volumetric flask in Methanol with 0.1N NH3 to a concentration of 200 ng/ml each (**A2**)
  - Primary Standards were diluted to two solutions

        - **B1** -- 10 ng/ul Stock Mix: 150 ul of both T3 and T4 diluted to 1.5 ml with Methanol + 0.1N NH3
        - **B2** -- 1 ng/ul Stock Mix: 100 ul of B1 diluted to 1 ml with Methanol + 0.1N NH3

Standard Calibration Curve:

- Working solutions of the Primary Standards were made at the following concentrations in Methanol + 0.1 N NH3: 5, 10, 25, 50, 100, 250, 500, 1000, 2500, 5000, 10,000 ng/ml from Stock solutions B1and B2
- Standard Calibration Curve Dilutions with ISTD
    - 20 ul of each working solution, 20 ul of ISTD A2 mix, and 180 BLK Media were added to 1. 5 ml microcentrifuge tubes.
    - 400 ul of Acetonitrile was added to each for final Standard Calibration Curve Concentrations of 0.5, 1.0, 2.5, 5, 10, 25, 50, 100, 250, 250, 500, and 1000 ng/ml
    - Quality Controls were made up similarly at 1.25, 12.5, and 125 ng/ml and Labeled QC-L, QC-M, and QC-H, respectively
    - Each Calibrant and QC was then vortexed for 1 min and centrifuged at 13,000 rpm for 10 min at 4C
    - The 500ul supernatant for each Calibrant and QC was moved to a fresh tube and the solvent evaporated under Nitrogen
    - The Calibrants and QCs were reconstituted with 200 ul 3:1 Water/Methanol
    - Vortex and centrifuged prior moving 180 ul to autosampler vials with inserts for analysis on the LC-MS/MS

Extraction Procedure for Samples:

- Place 200 ul with 20 ul ISTD A2 solution in a 1.5 ml microcentrifuge tube
- Add 400 ul of Acetonitrile is add to tube
- Vortex for 1 min
- Centrifuge at 13,000 rpm for 10 minutes at 4°C
- Transfer 500 ul of supernatant was moved to a fresh tube and the solvent evaporated under Nitrogen
- Samples were reconstituted with 200 ul 3:1 Water/Methanol
- Vortex and centrifuged prior moving 180 ul to autosampler vials with inserts for analysis on the LC-MS/MS.

Data Collection and Processing

- The LC-MS/MS data are acquired using a Waters Xevo TQ-S cronos triple quadrupole mass spectrometer coupled to Waters Acquity H-Class UPLC system

Liquid Chromatography Method:

- LC Column: Waters Acquity Premier BEH C18 (2.1 x 50 mm, 1.8 um) with inline filter
- Mobile phase was comprised of:
- Solvent A: Water with 0.1% acetic Acid and
- Solvent B: Methanol.
- Column temperature: 50°C

- Flow rate: 0.5 ml/min
- Injection Volume: 10 ul
- Chromatographic gradient:
  - The gradient starts at 40% Solvent B, which is held for 0.5 min
  - Then increased linearly to 98% Solvent B over 4.0 min
  - It is held at 98% Solvent B for 0.5 min
  - Then returned to 40% Solvent B in 0.1 min and equilibrated until 7.0 min

Mass Spectrometer Parameters:

- The TQ-S mass spectrometer was operated in positive polarity mode, with the spray voltage set to 3.0 kV, the ion source temperature at 150°C, and the desolvation gas temperature held at 400°C.
- The desolvation gas flow was set to 900 L/Hr and the Cone Gas flow was 50 L/Hr.
- MS data was collected using multiple reaction monitoring (MRM) mode.
- The three MRM transitions for [(M+H)+ ion to product ions] used for quantification of T4 were as follows: 777.46→196.55, 777.46→323.19, and 777.46→731.41. Cone voltage of 60V and Collision Energies of 66, 64, and 22 eV, respectively.
- For T3 the MRM transition used were: 651.57→197, 651.57→507.62, and 651.57→605.54. Cone voltage of 50V and Collision Energies of 68, 24, and 22 eV, respectively.
- The following MRM were used for T4-13C6: 783.71→737.40 and for T3-13C6: 657.82→611.70 with Cone voltages of 60 and 50 V and Collision Energies of 24eV and 22eV, respectively.

Data Processing

- Waters MassLynx 4.2 software was used for data acquisition and initial data quality control.
- Further data processing performed with Waters TargetLynx for peak detection and quantification.
- Data after processing in TargetLynx was transferred to Excel sheet for drawing the calibration curve and calculate the concentrations. The amount calculated from calibration curve for terazosin in each sample should be multiply to 1.24 (1/[200 µl samples / (400 µl extraction volume buffer + 20 µl ISTD solution + 200 µl sample)] x [500 µl extraction used for analysis / 200 µl resuspension volume]) for the exact amount in 200 µl of started media with.
