# Supplementary material for: Human Neutrophils Produce De Novo Thyroid Hormones Mediated by the Induction of the Oxidative Burst
Source: Biomolecules. 2026 Jul 23;16(8):1081. doi: 10.3390/biom16081081 (PMC13511619; doi:10.3390/biom16081081)

Supp Figure S1

A

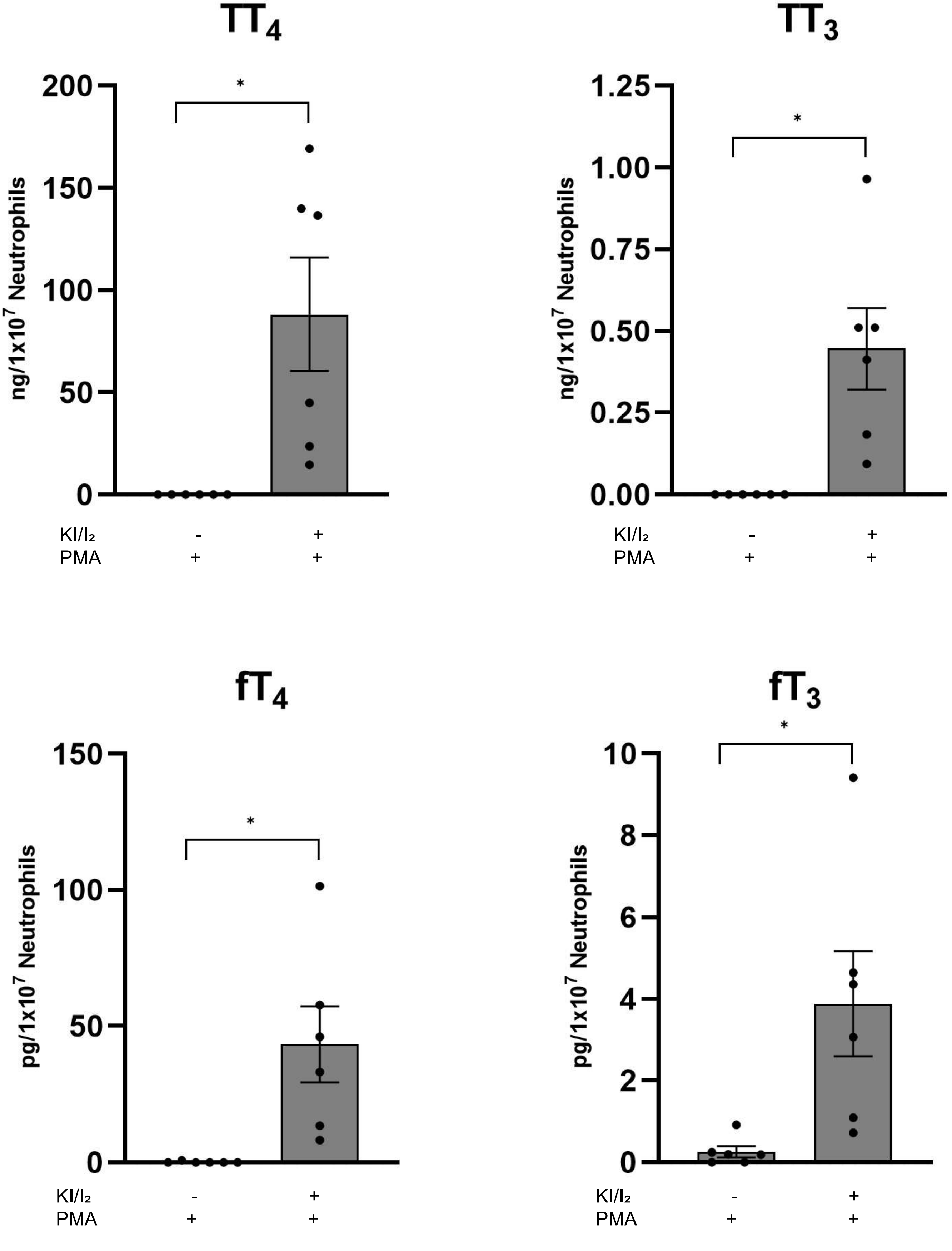

Supplement: Supplementary file 1 [file biomolecules-16-01081-s001.zip › Supp Figure S1.pdf]
